# The landscape of 605 genetically confirmed distinct rare diseases in a single center in Mexico (2005–2025)

**DOI:** 10.1186/s13023-026-04318-1

**Published:** 2026-03-20

**Authors:** Juan Carlos Zenteno, Vianey Ordoñez-Labastida, Luis Montes-Almanza, Froylan Garcia-Martinez, Alejandro Martinez-Herrera, David Carreño-Bolaños, Rocio Arce-Gonzalez, Oscar F. Chacón-Camacho

**Affiliations:** 1 Rare Disease Diagnostic Unit, Faculty of Medicine, National Autonomous University of Mexico (UNAM), Mexico City, Mexico; 2 Department of Genetics, Institute of Ophthalmology “Conde de Valenciana”, Mexico City, Mexico; 3 Biochemistry Department, Faculty of Medicine, UNAM, Mexico City, Mexico; 4 Faculty of Medicine, Autonomous University of the State of Morelos, Cuernavaca, Morelos Mexico; 5 Laboratorio 5, Edificio A-4, Carrera de Médico Cirujano, Facultad de Estudios Superiores Iztacala, UNAM, Mexico City, Mexico; 6 Department of Genetics, Institute of Ophthalmology “Conde de Valenciana, ” Chimalpopoca 14, Col. Obrera, Cuauhtemoc, Mexico City, CP06800 Mexico

**Keywords:** Rare diseases, Registry, Genetic testing, Mexico

## Abstract

**Background:**

Timely diagnosis and therapeutic management of rare diseases (RDs) are greatly hampered by limited clinical information and a lack of reliable epidemiological data. RDs constitute a heterogeneous group of disorders, and although they have become major public health issues, there is a paucity of both population- and disease-based data on them, particularly in developing countries. Next-generation sequencing (NGS) has revolutionized the diagnosis of these disorders by allowing fast and cost-effective identification of disease-causing genetic variants, as well as the development of reliable, molecular diagnosis–based registries. Presented here is a 20-year retrospective, single-center study of molecularly confirmed RDs, with causal variants classified as pathogenic or likely pathogenic according to the ACMG criteria. Only disorders with an OMIM disease number or publications regarding them were considered.

**Results:**

A total of 605 RDs, classified in 14 different clinical categories, were genetically confirmed. The most common diseases were ocular disorders (34%), neurodevelopmental disorders (23.5%), and neurological disorders (11%). Among the 605 identified disorders, 326 were autosomal dominant, 225 were autosomal recessive, 43 were X-linked, 7 had mitochondrial inheritance, and 4 were caused by somatic mosaicism. A total of 534 genes/genomic regions were demonstrated to carry disease-causing variants.

**Conclusion:**

This is the first catalog of genetically confirmed RDs in Mexico, and it can be considered a first step toward a better characterization of the spectrum of such diseases in the country. Patients’ increasing access to diagnostic exome and/or genome sequencing tests has enabled unprecedented success in RD characterization at the genetic level. The vast majority of genetic diagnoses in our series (~ 75%) occurred in the past eight years, which coincides with a wider availability of NGS testing in Mexico. Our catalog is freely accessible through an online database (https://0198bb3b-526c-b682-3f6d-c5894015db9b.share.connect.posit.cloud/), which is intended to be continuously maintained, extended, and updated with contributions from other health professionals in the field of RDs in Mexico. This resource might accelerate the characterization of such diseases in the country, and it may also play a role in the development of a reliable national registry of RD patients.

**Supplementary Information:**

The online version contains supplementary material available at 10.1186/s13023-026-04318-1.

## Background

Rare diseases (RDs) are defined as conditions occurring with a prevalence of fewer than 1 in 2.000 individuals in the general population. To date, between 8.000 and 10.000 different RDs have been identified, which collectively affect 400 million people worldwide [1]. RDs are associated with significant diagnostic delay, high morbidity and mortality, elevated diagnostic and management costs, and lack of access to treatments [2]. It is estimated that up to 80% of RDs are of genetic origin, with the remaining arising from rare infections, autoimmune diseases, or atypical neoplasia, among other causes [3].

Although RDs have been recognized as a major public health issue, there is a paucity of population-based data and epidemiological information on them in most countries. The advent of massive DNA sequencing techniques for the diagnosis of genetic disorders has revolutionized the field of RDs by allowing definitive diagnoses in thousands of patients; this has shortened their diagnostic odysseys and enabled more rational and individualized medical management [4–8]. Several studies have shown the benefits of early diagnosis in RD patients, not only in terms of medical care but also of health system efficiency [9–12].

RD registries, cohorts, and databases are valuable instruments for planning clinical research and, ultimately, improving health care for RD patients, who are frequently marginalized [13, 14]. However, many patients with suspected RDs are diagnosed based merely on clinical features, which frequently leads to incorrect diagnoses due to the extensive overlap between the clinical manifestations of different RDs. It has been estimated that between 20% and 50% of clinically assigned diagnoses are modified after molecular testing [15–18]. Thus, genetic testing for definitive diagnosis is a central part of reliable RD registries worldwide.

With a population exceeding 130 million, Mexico is the 10th-most populous country in the world. According to official figures, at least eight million individuals have a rare disease in the country [19]. Despite this, no published registries exist on the types of RDs occurring in the country, nor on the molecular spectrum associated with these disorders in Mexicans. The Mexican population is mainly composed of mestizos, who are individuals with a genetic background consisting of Amerindian, European, and, to a lesser extent, African ancestries.

Here, we present a catalog of molecularly confirmed genetic RDs identified over a 20-year period in patients from a single center in Mexico. More than 600 individual conditions were recognized, which highlights the heterogeneity of RDs and paints an initial picture of their occurrence in the country. This catalog will be made available on a dedicated, freely accessible website, and it will be continually updated with information from other RD centers, with the ultimate goal of characterizing a more complete profile of RDs in Mexico.

## Methods

### Design

A retrospective study was conducted in the Rare Disease Diagnostic Unit (UDER)-and the Genetics Department of the Institute of Ophthalmology “Conde de Valenciana,” a single center that is part of the Faculty of Medicine of the National Autonomous University of Mexico in Mexico City. This center is a national referral center for genetic diagnosis, and it consists of two testing facilities: the genetic eye disease diagnostic laboratory and the general (nonocular) rare disease diagnostic unit. While patients from all over the country are referred to these facilities and evaluated there, most of them come from Mexico City and other states in central Mexico. In both facilities, board-certified geneticists are responsible for the clinical evaluation of patients and genetic testing indication.

Only diseases confirmed through genetic testing between January 2005 and July 2025 were registered in the catalog. Genetic analysis methods comprised Sanger sequencing, SNPs microarrays, and massive DNA sequencing techniques, including gene panel, exome sequencing, and genome sequencing. Genetic testing has evolved from single-gene analysis to comprehensive genomic profiling; today, a combination of techniques is employed for genetic diagnosis. In our cohort, Sanger sequencing was the most commonly employed technique starting in 2005. Gene panel sequencing was introduced in 2014, followed by exome sequencing in 2017. Bioinformatic analysis for copy number variations (CNVs) identification was introduced in 2021. Data from negative next-generation sequencing (NGS) studies are reanalyzed each year for possible reclassification of variants of unknown significance (VUSs) into likely pathogenic or pathogenic variants.

For CNV (deletions/duplications spanning at least one gene exon) detection, ExomeDepth, an R package for the efficient detection of copy number variants in exomes and gene panels that uses high-throughput DNA sequencing data, was employed. Clinically relevant CNVs identified by NGS were confirmed using quantitative PCR. Suspected mosaicism and somatic variations were investigated by Sanger sequencing of additional tissue samples (buccal cells and skin), when available.

### Definition of genetic diagnosis

A genetic diagnosis was considered positive upon identification of a variant classified as pathogenic or likely pathogenic according to the ACMG criteria [20] in a known gene or chromosome locus that explained the patient’s phenotype and occurred with the expected zygosity (i.e., monoallelic for dominant conditions, biallelic for recessive disorders, and hemizygous in X-linked recessive diseases). Cases with only VUSs or without candidate disease-causing variants were not included.

### Criteria for disease inclusion

Only diseases with an OMIM code or published academic papers regarding them were considered. Each diagnosis was counted once, regardless of how many patients presented with the same condition. Disease categories were recorded following the WHO’s International Classification of Diseases (eleventh revision). Accordingly, the neurodevelopmental disorder category was defined as a group of conditions with onset in the developmental period, and it included intellectual disability, communication disorders, autism spectrum disorder, attention deficit hyperactivity disorder, neurodevelopmental motor disorders, and learning disorders. Neurological disorders are conditions that affect the functions of the central and peripheral nervous systems.

Complex phenotypes were excluded from genetic analysis if a history of maternal exposure to potential teratogenic agents was recorded.

## Results

A total of 2,579 patients were identified in our study, including 1,342 females (52%) and 1,237 males (48%). Age at genetic testing ranged from two weeks to 98 years, with a mean of 25.3 years. Regarding geographic origin, most patients came from the central region of Mexico, with Mexico City the most frequent site (35%), followed by Hidalgo (7%) and Puebla (5.5%) states.

**Fig. 1:**
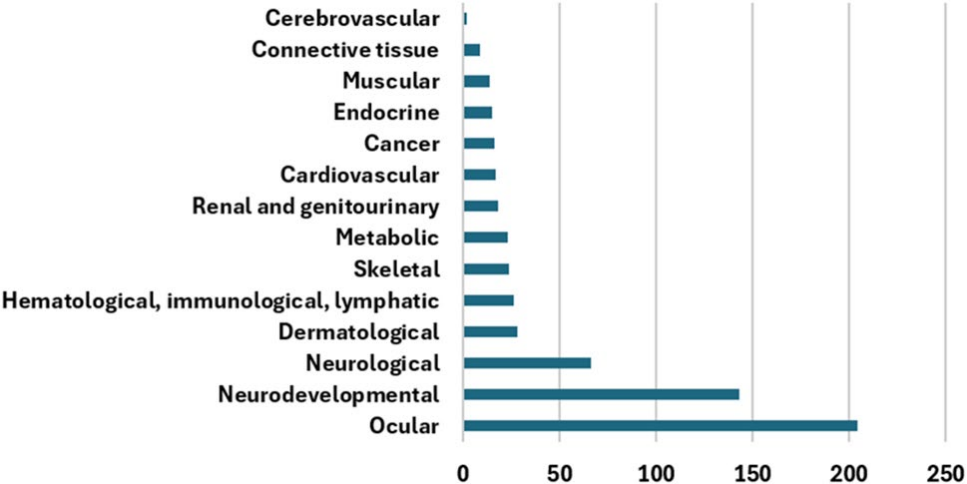
Bar graph showing the number of affected cases in each major disease category in this study

A total of 605 genetic RDs were identified in our retrospective cohort. As shown in Fig. 1 and Table 1, 14 disease categories were recorded. For each individual disorder, at least one patient with a causative pathogenic variant (or variants) was identified (see Supplementary Table 1).

**Table 1:** Disease category frequencies and numbers of genes/loci carrying disease-causing variants among the 605 RDs identified in the study

Disease category	*n* (%)	Involved genes/loci
Ocular	205 (33.9%)	181
Neurodevelopmental	142 (23.4%)	140
Neurological	66 (11%)	62
Dermatological	28 (4.6%)	26
Hematologic, immunological, and lymphatic	26 (4.3%)	25
Skeletal	24 (4%)	21
Metabolic	23 (3.8%)	23
Renal and genitourinary	18 (3%)	18
Cardiovascular	17 (2.8%)	15
Cancer	16 (2.6%)	15
Endocrine	15 (2.4%)	15
Muscular	14 (2.3%)	13
Connective tissue	9 (1.5%)	8
Cerebrovascular	2 (0.4%)	2
**TOTAL**	**605 (100%)**	**564**

The five largest disease categories in our registry were ocular disorders (*n* = 204; 34%), neurodevelopmental disorders (*n* = 143; 23.5%), neurological disorders (*n* = 66; 11%), dermatological diseases (*n* = 28; 4.7%), and hematologic, immunological, and lymphatic disorders (*n* = 26; 4.3%). The most common genetically confirmed diagnoses were retinal dystrophies, anomalies of the anterior segment of the eye, and developmental and epileptic encephalopathies.

**Fig. 2:**
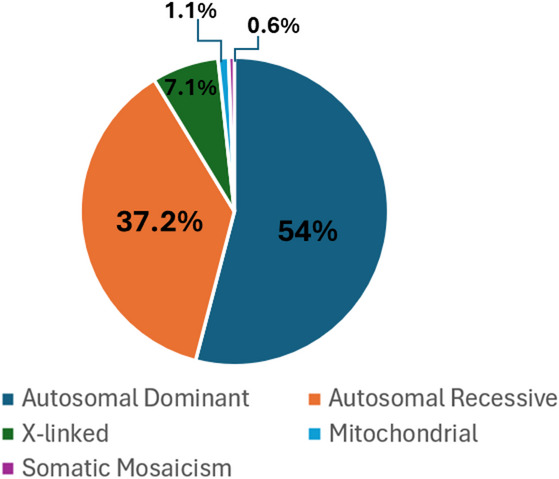
Pie graph showing the modes of inheritance proportions observed in 605 different rare diseases in this study

Based on the inheritance patterns (Fig. 2; Table 2), 326 autosomal dominant, 225 autosomal recessive, and 43 X-linked diseases were identified. In addition, 7 disorders with mitochondrial inheritance and 4 diseases due to somatic mosaicism were found. Sixteen genetic disorders caused by segmental aneusomies (submicroscopic deletions/duplications) were identified in the cohort.

**Table 2:** Inheritance pattern frequencies among the 605 RDs identified in the study

Inheritance pattern	*n*	%
Autosomal dominant	326	53.8%
Autosomal recessive	225	37.2%
X-linked	43	7.1%
Mitochondrial	7	1.2%
Somatic mosaicism	4	0.7%
**Total**	**605**	**100**

While the objective of this work was not to establish the number of patients with individual RDs diagnosed at our institution, the commonest genetically confirmed diagnoses included Stargardt disease, retinitis pigmentosa, Usher syndrome, and oculo-pharyngeal muscular dystrophy. These data reflect a bias of the present study, which was conducted at a referral ophthalmologic hospital. This fact meant that inherited ocular diseases were the most frequently genetically diagnosed disorders at our center.

Due to the occurrence of allelic disorders (i.e., different diseases arising from mutations in the same gene/locus), the total number of mutated genes/loci was 534 resulting in 605 different phenotypes, as mentioned above (Supplementary Table 1). The mutated genes with the greatest number of different phenotypes were *TGFBI* (5 phenotypes), *PROM1* (4 phenotypes), *BEST1* (4 phenotypes), *TULP1* (3 phenotypes), and *KRAS* (3 phenotypes). Notably, 29 out of the 605 diseases currently lack an OMIM code, including 14 autosomal dominant, 13 autosomal recessive, and 2 X-liked disorders; however, papers regarding them have been published in peer-reviewed academic journals. Remarkably, over 75% of the genetic diagnoses were made during the past eight years (2018–2025), reflecting the increasing availability of massive genetic sequencing tests for RD diagnosis. Supplementary table S1 alphabetically lists all the genes with confirmed disease-causing variants, the names of the associated diseases, their individual OMIM codes, and their inheritance patterns.

## Discussion

The heterogeneity of RDs, with their diverse and overlapping symptoms, complicates the diagnostic process. This clinical heterogeneity requires specialized diagnostic tests and genetic screening to confirm suspected diagnoses. Despite great advances in diagnostics and research, many RD patients continue to live without a definitive genetic diagnosis. Currently, the most commonly employed RD genetic diagnostic methods are exome and genome sequencing, which have diagnostic yields ranging between 20% and 70%, depending on the category of the disease and the inclusion criteria, among other factors [21–23]. Here, we present the diagnostic landscape of a large cohort of individuals with RDs in a single center in Mexico for a period of 20 years. A total of 605 RDs were genetically confirmed, illustrating the heterogeneity of these diseases and the value of diagnostic genetic testing. Among the 14 clinical categories, the most frequent diseases were ocular disorders (34%), neurodevelopmental disorders (23.5%), neurological disorders (11%), dermatological diseases (~ 5%), and hematologic, immunological, and lymphatic disorders (~ 4%).

In our study, neurodevelopmental disorders accounted for a quarter of genetically confirmed diseases. Neurodevelopmental disorders are commonly identified among diseases with higher NGS-diagnostic rates [8, 22, 24, 25]. This high diagnostic rate may be related to the fact that the ACMG guidelines suggest that patients with this diagnosis should be genetically tested, with exome sequencing currently being the first diagnostic testing option.

The lack of epidemiological data on RDs, or the presence of inaccurate data, is a problem across the world. Therefore, the creation of disease catalogs and/or patient registries is imperative to better profile these disorders among populations and identify their geographical variations. According to the Orphanet database, an international initiative providing valuable resources for RDs, there are currently more than 800 established registries, databases, and cohorts, most of which are based in developed countries [13]. However, catalogs of the occurrence of RDs are practically nonexistent in many developing countries. The characterization of over 600 RDs through DNA analysis testing in the present single-center registry clearly indicates the importance of genetic testing in subjects with suspected RDs. This type of testing is particularly significant to construct trustworthy registries of RDs based on more than patients’ clinical features. While it is estimated that the number of RDs could reach 10,000, information about the number of confirmed individual diseases in countries or ethnic groups is unavailable. For example, India has reported about 450 types of RDs, according to the database of the Indian Organization for Rare Diseases [26].

Patients’ increasing access to diagnostic exome and/or genome sequencing tests has allowed unprecedented success in RD characterization at the genetic level. The great majority of identified diagnoses in our series (~ 75%) occurred in the past eight years, which coincides with a wider availability of NGS testing in Mexico. Still, as in many other countries, several challenges and obstacles remain that prevent a broader application of genetic testing to RD patients [27, 28]. The main problem is that genetic and genomic tests are not subsidized by the Mexican public health system; hence, patients are required to pay the full cost of DNA testing. In addition, sparse genetic services centers in the country result in a lack of clinical and molecular evaluation for most RD patients.

Although our study was based on a cohort of over 2,500 patients, and more than 600 RDs were identified as part of it, several biases need to be recognized. First of all, our facility is part of an ophthalmic referral center; thus, ocular disorders ranked first in our diagnostic series. In addition, cases with only VUSs or without candidate disease-causing variants were not included in the registry, but several of them could be diagnosed at the clinical level. Moreover, our data cannot be used to infer prevalence or diagnostic yield at the population level. Finally, given the unequal application of genetic tests to our cohort over time, a confident diagnostic yield cannot be calculated from our data.

## Conclusion

This is the first catalog of genetically confirmed RDs in Mexico, and it can be considered a first attempt to characterize the spectrum of such diseases in the country. Using the Shiny web framework, we have developed an online application to facilitate the distribution of our data (https://0198bb3b-526c-b682-3f6d-c5894015db9b.share.connect.posit.cloud/). The online database includes statistics on disease categories and distribution, as well as inheritance pattern frequencies; it also includes a table with the 605 identified diseases and their respective linkable OMIM disease numbers or relevant references. This freely accessible online catalog is intended to be maintained, extended, and updated monthly with data from other health professionals working in the field of RDs in Mexico. This resource will accelerate the characterization of RDs occurring in the country, and it may also contribute to a reliable national registry of RD patients.

## Supplementary Information

Below is the link to the electronic supplementary material.

Supplementary Material 1

Supplementary Material 2

## Data Availability

The dataset supporting the conclusions of this article is included in the article and its supplementary file. Additional data may be made available on request to the corresponding author.

## Abbreviations

ACMG: American College of Medical Genetics and Genomics
DNA: Desoxyribonucleic acid
NGS: Next-generation sequencing
OMIM: Online Mendelian Inheritance in Man
RD: Rare disease
SNPs: Single-nucleotide polymorphism
UDER: Rare Disease Diagnostic Unit
UNAM: National Autonomous University of Mexico
