# Supplementary material for: The landscape of 605 genetically confirmed distinct rare diseases in a single center in Mexico (2005–2025)
Source: Orphanet J Rare Dis. 2026 Mar 20;21:170. doi: 10.1186/s13023-026-04318-1 (PMC13126887; doi:10.1186/s13023-026-04318-1)
Supplement: Supplementary file 1 — Supplementary Material 1 [file 13023_2026_4318_MOESM1_ESM.docx]

**Suppl. table #1 references**

Allach El Khattabi L, Heide S, Caberg JH, Andrieux J, Doco Fenzy M, Vincent-Delorme C, Callier P, Chantot-Bastaraud S, Afenjar A, Boute-Benejean O, Cordier MP, Faivre L, Francannet C, Gerard M, Goldenberg A, Masurel-Paulet A, Mosca-Boidron AL, Marle N, Moncla A, Le Meur N, Mathieu-Dramard M, Plessis G, Lesca G, Rossi M, Edery P, Delahaye-Duriez A, De Pontual L, Tabet AC, Lebbar A, Suiro L, Ioos C, Natiq A, Chafai Elalaoui S, Missirian C, Receveur A, François-Fiquet C, Garnier P, Yardin C, Laroche C, Vago P, Sanlaville D, Dupont JM, Benzacken B, Pipiras E. 16p13.11 microduplication in 45 new patients: refined clinical significance and genotype-phenotype correlations. J Med Genet. 2020 May;57(5):301-307.

Aldrees A, Abdelkader E, Al-Habboubi H, Alrwebah H, Rahbeeni Z, Schatz P. Non-syndromic retinal dystrophy associated with homozygous mutations in the ALMS1 gene. Ophthalmic Genet. 2019 Feb;40(1):77-79.

Astiazarán MC, García-Montaño LA, Sánchez-Moreno F, Matiz-Moreno H, Zenteno JC. Next generation sequencing-based molecular diagnosis in familial congenital cataract expands the mutational spectrum in known congenital cataract genes. Am J Med Genet A. 2018 Dec;176(12):2637-2645.

Ayala-Ramirez R, Graue-Wiechers F, Robredo V, Amato-Almanza M, Horta-Diez I, Zenteno JC. A new autosomal recessive syndrome consisting of posterior microphthalmos, retinitis pigmentosa, foveoschisis, and optic disc drusen is caused by a MFRP gene mutation. Mol Vis. 2006 Dec 4;12:1483-9.

Borroto MC, Campeau PM, et al. A Genotype/Phenotype Study of KDM5B-Associated Disorders Suggests a Pathogenic Effect of Dominantly Inherited Missense Variants. Genes (Basel). 2024 Aug 6;15(8):1033.

Boulanger-Scemama E, El Shamieh S, Démontant V, Condroyer C, Antonio A, Michiels C, Boyard F, Saraiva JP, Letexier M, Souied E, Mohand-Saïd S, Sahel JA, Zeitz C, Audo I. Next-generation sequencing applied to a large French cone and cone-rod dystrophy cohort: mutation spectrum and new genotype-phenotype correlation. Orphanet J Rare Dis. 2015 Jun 24;10:85.

Coussa RG, Otto EA, Gee HY, Arthurs P, Ren H, Lopez I, Keser V, Fu Q, Faingold R, Khan A, Schwartzentruber J, Majewski J, Hildebrandt F, Koenekoop RK. WDR19: an ancient, retrograde, intraflagellar ciliary protein is mutated in autosomal recessive retinitis pigmentosa and in Senior-Loken syndrome. Clin Genet. 2013 Aug;84(2):150-9.

Dalma-Weiszhausz J, Chacón-Camacho O, Chevez-Barrios P, Zenteno JC, Franco-Cárdenas V, García-Montaño LA, Pérez-Bravo J, García-Montalvo IA, Jiménez-Sierra JM, Dalma A. AUTOSOMAL DOMINANT MÜLLER CELL SHEEN DYSTROPHY: Clinical, Histopathologic, and Genetic Assessment in an Extended Family With Long Follow-Up. Retina. 2022 May 1;42(5):981-991.

El Hayek L, Tuncay IO, Nijem N, Russell J, Ludwig S, Kaur K, Li X, Anderton P, Tang M, Gerard A, Heinze A, Zacher P, Alsaif HS, Rad A, Hassanpour K, Abbaszadegan MR, Washington C, DuPont BR, Louie RJ; CAUSES Study; Couse M, Faden M, Rogers RC, Abou Jamra R, Elias ER, Maroofian R, Houlden H, Lehman A, Beutler B, Chahrour MH. *KDM5A* mutations identified in autism spectrum disorder using forward genetics. Elife. 2020 Dec 22;9:e56883.

Genovese A, Cox DM, Butler MG. Partial Deletion of Chromosome 1p31.1 Including only the Neuronal Growth Regulator 1 Gene in Two Siblings. J Pediatr Genet. 2015 Mar;4(1):23-8. doi: 10.1055/s-0035-1554977.

Hisama FM, Dib-Hajj SD, Waxman SG. SCN9A Neuropathic Pain Syndromes. 2006 May 6 [Updated 2020 Jan 23]. In: Adam MP, Feldman J, Mirzaa GM, et al., editors. GeneReviews® [Internet]. Seattle (WA): University of Washington, Seattle; 1993-2025. Available from: <https://www.ncbi.nlm.nih.gov/books/NBK1163/>

Hu M, Li H, Huang Z, Li D, et al. Novel compound heterozygous mutation in STAMBP causes a neurodevelopmental disorder by disrupting cortical proliferation. Front Neurosci. 2022 Aug 10;16:963813.

Huang XF, Xiang L, Fang XL, Liu WQ, Zhuang YY, Chen ZJ, Shen RJ, Cheng W, Han RY, Zheng SS, Chen XJ, Liu X, Jin ZB. Functional characterization of CEP250 variant identified in nonsyndromic retinitis pigmentosa. Hum Mutat. 2019 Aug;40(8):1039-1045.

Lyons MJ. MED12-Related Disorders. 2008 Jun 23 [Updated 2021 Aug 12]. In: Adam MP, Feldman J, Mirzaa GM, et al., editors. GeneReviews® [Internet]. Seattle (WA): University of Washington, Seattle; 1993-2025. Available from: <https://www.ncbi.nlm.nih.gov/books/NBK1676/>

Ma W, Mao J, Wang X, Duan L, Song Y, Lian X, Zheng J, Liu Z, Nie M, Wu X. Novel Microdeletion in the X Chromosome Leads to Kallmann Syndrome, Ichthyosis, Obesity, and Strabismus. Front Genet. 2020 Jun 24;11:596. doi: 10.3389/fgene.2020.00596. PMID: 32670353; PMCID: PMC7327112.

Mantere T, Winqvist R, Kauppila S, Grip M, Jukkola-Vuorinen A, Tervasmäki A, Rapakko K, Pylkäs K. Targeted Next-Generation Sequencing Identifies a Recurrent Mutation in MCPH1 Associating with Hereditary Breast Cancer Susceptibility. PLoS Genet. 2016 Jan 28;12(1):e1005816.

Nishiguchi KM, Avila-Fernandez A, van Huet RA, Corton M, Pérez-Carro R, Martín-Garrido E, López-Molina MI, Blanco-Kelly F, Hoefsloot LH, van Zelst-Stams WA, García-Ruiz PJ, Del Val J, Di Gioia SA, Klevering BJ, van de Warrenburg BP, Vazquez C, Cremers FP, García-Sandoval B, Hoyng CB, Collin RW, Rivolta C, Ayuso C. Exome sequencing extends the phenotypic spectrum for ABHD12 mutations: from syndromic to nonsyndromic retinal degeneration. Ophthalmology. 2014 Aug;121(8):1620-7.

Overhoff J, Rabideau MM, Bird LM, Schweitzer DN, Haynes K, Schultz RA, Shaffer LG, Rosenfeld JA, Ellison JW. Refinement of the 8q22.1 microdeletion critical region associated with Nablus mask-like facial syndrome. Am J Med Genet A. 2014 Jan;164A(1):259-63. doi: 10.1002/ajmg.a.36163

Palumbo O, Palumbo P, Palladino T, Stallone R, Zelante L, Carella M. A novel deletion in 2q24.1q24.2 in a girl with mental retardation and generalized hypotonia: a case report. Mol Cytogenet. 2012 Jan 3;5(1):1

Plaisier E, Ronco P. COL4A1-Related Disorders. 2009 Jun 25 [Updated 2016 Jul 7]. In: Adam MP, Feldman J, Mirzaa GM, et al., editors. GeneReviews® [Internet]. Seattle (WA): University of Washington, Seattle; 1993-2025. Available from: <https://www.ncbi.nlm.nih.gov/books/NBK7046/>

Quinodoz M, Rodenburg K, Cvackova Z, Kaminska K, de Bruijn SE, Iglesias-Romero AB, Boonen EGM, Ullah M, Zomer N, Folcher M, Bijon J, Holtes LK, Tsang SH, Corradi Z, Freund KB, Shliaga S, Panneman DM, Hitti-Malin RJ, Ali M, AlTalbishi A, Andréasson S, Ansari G, Arno G, Astuti GDN, Ayuso C, Ayyagari R, Banfi S, Banin E, Barboni MTS, Bauwens M, Ben-Yosef T, Birch DG, Biswas P, Blanco-Kelly F, Bocquet B, Boon CJF, Branham K, Britten-Jones AC, Bujakowska KM, Cadena EL, Calzetti G, Cancellieri F, Cattaneo L, Issa PC, Chadderton N, Coutinho-Santos L, Daiger SP, De Baere E, de la Cerda B, De Roach JN, De Zaeytijd J, Derks R, Dhaenens CM, Dudakova L, Duncan JL, Farrar GJ, Feltgen N, Fernández-Caballero L, Sallum JMF, Gana S, Garanto A, Gardner JC, Gilissen C, Goto K, Gonzàlez-Duarte R, Griffiths-Jones S, Haack TB, Haer-Wigman L, Hardcastle AJ, Hayashi T, Héon E, Hoischen A, Holtan JP, Hoyng CB, Ibanez MBB 4th, Inglehearn CF, Iwata T, Jones K, Kalatzis V, Kamakari S, Karali M, Kellner U, Knézy K, Klaver CCW, Koenekoop RK, Kohl S, Kominami T, Kühlewein L, Lamey TM, Leroy BP, Martín-Gutiérrez MP, Martins N, Mauring L, Leibu R, Lin S, Liskova P, Lopez I, López-Rodríguez VRJ, Mahroo OA, Manes G, McKibbin M, McLaren TL, Meunier I, Michaelides M, Millán JM, Mizobuchi K, Mukherjee R, Nagy ZZ, Neveling K, Ołdak M, Oorsprong M, Pan Y, Papachristou A, Percesepe A, Pfau M, Pierce EA, Place E, Ramesar R, Rasquin FA, Rice GI, Roberts L, Rodríguez-Hidalgo M, Ruiz-Eddera J, Sabir AH, Sajiki AF, Sánchez-Barbero AI, Sarma AS, Sangermano R, Santos CM, Scarpato M, Scholl HPN, Sharon D, Signorini SG, Simonelli F, Sousa AB, Stefaniotou M, Stingl K, Suga A, Sullivan LS, Szabó V, Szaflik JP, Taurina G, Toomes C, Tran VH, Tsilimbaris MK, Tsoka P, Vaclavik V, Vajter M, Valeina S, Valente EM, Valentine C, Valero R, van Aerschot J, van den Born LI, Webster AR, Whelan L, Wissinger B, Yioti GG, Yoshitake K, Zenteno JC, Zeuli R, Zuleger T, Landau C, Jacob AI, Cremers FPM, Lee W, Ellingford JM, Stanek D, Rivolta C, Roosing S. De novo and inherited dominant variants in U4 and U6 snRNAs cause retinitis pigmentosa. medRxiv [Preprint]. 2025 Jan 6:2025.01.06.24317169. doi: 10.1101/2025.01.06.24317169.

Reis LM, Maheshwari M, Capasso J, Atilla H, Dudakova L, Thompson S, et al. Axenfeld-Rieger syndrome: more than meets the eye. J Med Genet. 2023 Apr;60(4):368-379.

Sangermano R, Fujinami K, Byeon SH, et al. Variants in the ciliopathy gene SCLT1 are associated with non-syndromic retinal degeneration, 19 May 2025, PREPRINT (Version 1) available at Research Square [https://doi.org/10.21203/rs.3.rs-6507107/v1]

Sarkar H, Dubis AM, Downes S, Moosajee M. Novel Heterozygous Deletion in Retinol Dehydrogenase 12 (*RDH12*) Causes Familial Autosomal Dominant Retinitis Pigmentosa. Front Genet. 2020 Apr 8;11:335.

Sheikh SA, Sisk RA, Schiavon CR, Waryah YM, Usmani MA, Steel DH, Sayer JA, Narsani AK, Hufnagel RB, Riazuddin S, Kahn RA, Waryah AM, Ahmed ZM. Homozygous Variant in ARL3 Causes Autosomal Recessive Cone Rod Dystrophy. Invest Ophthalmol Vis Sci. 2019 ;60(14):4811-4819.

Testa F, Sodi A, Signorini S, Di Iorio V, Murro V, Brunetti-Pierri R, Valente EM, Karali M, Melillo P, Banfi S, Simonelli F. Spectrum of Disease Severity in Nonsyndromic Patients With Mutations in the CEP290 Gene: A Multicentric Longitudinal Study. Invest Ophthalmol Vis Sci. 2021 Jul 1;62(9):1.

Wang F, Wang H, Tuan HF, Nguyen DH, Sun V, Keser V, Bowne SJ, Sullivan LS, Luo H, Zhao L, Wang X, Zaneveld JE, Salvo JS, Siddiqui S, Mao L, Wheaton DK, Birch DG, Branham KE, Heckenlively JR, Wen C, Flagg K, Ferreyra H, Pei J, Khan A, Ren H, Wang K, Lopez I, Qamar R, Zenteno JC, Ayala-Ramirez R, Buentello-Volante B, Fu Q, Simpson DA, Li Y, Sui R, Silvestri G, Daiger SP, Koenekoop RK, Zhang K, Chen R. Next generation sequencing-based molecular diagnosis of retinitis pigmentosa: identification of a novel genotype-phenotype correlation and clinical refinements. Hum Genet. 2014 Mar;133(3):331-45.

Zahid, S. *et al.* (2018). *ARL6/BBS3* . In: Retinal Dystrophy Gene Atlas. Springer, Cham. https://doi.org/10.1007/978-3-319-10867-4_4

Zenteno JC, Buentello-Volante B, Ayala-Ramirez R, Villanueva-Mendoza C. Homozygosity mapping identifies the Crumbs homologue 1 (Crb1) gene as responsible for a recessive syndrome of retinitis pigmentosa and nanophthalmos. Am J Med Genet A. 2011 May;155A(5):1001-6.
