# Supplementary material for: The landscape of 605 genetically confirmed distinct rare diseases in a single center in Mexico (2005–2025)
Source: Orphanet J Rare Dis. 2026 Mar 20;21:170. doi: 10.1186/s13023-026-04318-1 (PMC13126887; doi:10.1186/s13023-026-04318-1)
Supplement: Supplementary file 2 — Supplementary Material 2 [file 13023_2026_4318_MOESM2_ESM.docx]

**Supplemetary table 1**.- Complete list of 605 RDs identified in our study indicating involved gene, OMIM number, disease category

and number of identified patients in the cohort.

| **# Gene/ Locus** | **Gene** | **# Disease** | **Disease** | **OMIM #** | **Disease category** | **Inheritance** | **References for diseases without OMIM code (see suppl file)** | **Number of patients identified in this study** |
| --- | --- | --- | --- | --- | --- | --- | --- | --- |
| **1** | *AAAS* | **1** | Achalasia-addisonianism-alacrimia syndrome | 231550 | Ocular | AR |  | 1 |
| **2** | *ABCA4* | **2** | Stargardt disease, type 1 | 248200 | Ocular | AR |  | 211 |
|  | *ABCA4* | **3** | Cone-rod dystrophy, type 3 | 604116 | Ocular | AR |  | 18 |
|  | *ABCA4* | **4** | Retinitis pigmentosa, type 19 | 601718 | Ocular | AR |  | 1 |
| **3** | *ABCB11* | **5** | Cholestasis, progressive familial intrahepatic, type 2 | 601847 | Metabolic | AR |  | 1 |
| **4** | *ABCC6* | **6** | Pseudoxanthoma elasticum | 264800 | Connective tissue | AR |  | 17 |
| **5** | *ABCD1* | **7** | Adrenoleukodystrophy | 300100 | Neurological | XL |  | 1 |
| **6** | *ABHD12* | **8** | Retinitis pigmentosa |  | Ocular | AR | **Nishiguchi et al., 2014** | 1 |
| **7** | *ACSF3* | **9** | Combined malonic and methylmalonic aciduria | 614265 | Metabolic | AR |  | 1 |
| **8** | *ACTA1* | **10** | Congenital myopathy type 2A, typical | 161800 | Muscular | AD |  | 1 |
| **9** | *ACTG1* | **11** | Baraitser-Winter sydrome, type 2 | 614583 | Neurodevelopmental | AD |  | 2 |
| **10** | *ACVR1* | **12** | Fibrodysplasia ossificans progressiva | 135100 | Skeletal | AD |  | 1 |
| **11** | *ACVRL1* | **13** | Telangiectasia, hereditary hemorrhagic, type 2 | 600376 | Cerebrovascular | AD |  | 2 |
| **12** | *ADA2* | **14** | Vasculitis, autoinflammation, immunodeficiency, and hematologic defects syndrome | 615688 | Hematological, Immunological and Lymphatic | AR |  | 2 |
| **13** | *ADAM9* | **15** | Cone-rod dystrophy, type 9 | 612775 | Ocular | AR |  | 1 |
| **14** | *ADAMTS17* | **16** | Weill-marchesani syndrome, type 4 | 613195 | Skeletal | AR |  | 1 |
| **15** | *ADAMTSL4* | **17** | Ectopia lentis et pupillae | 225200 | Ocular | AR |  | 1 |
| **16** | *ADGRL1* | **18** | Developmental delay, behavioral abnormalities, and neuropsychiatric disorders | 620065 | Neurodevelopmental | AD |  | 1 |
| **17** | *ADGRV1* | **19** | Usher syndrome, type 2C | 605472 | Ocular | AR |  | 3 |
| **18** | *ADSL* | **20** | Adenylosuccinase deficiency | 103050 | Metabolic | AR |  | 1 |
| **19** | *AGRN* | **21** | Myasthenic syndrome, congenital, type 8, with pre- and postsynaptic defects | 615120 | Neurological | AR |  | 1 |
| **20** | *AHDC1* | **22** | Xia-Gibbs syndrome | 615829 | Neurodevelopmental | AD |  | 2 |
| **21** | *AHI1* | **23** | Joubert syndrome, type 3 | 608629 | Neurodevelopmental | AR |  | 4 |
| **22** | *AIPL1* | **24** | Leber congenital amaurosis, type 4 | 604393 | Ocular | AR |  | 5 |
| **23** | *AIRE* | **25** | Autoinmune polyendocrinopathy syndrome, type I, with or without reversible mataphyseal dysplasia | 240300 | Hematological, Immunological and Lymphatic | AD |  | 2 |
| **24** | *ALMS1* | **26** | Alstrom syndrome | 203800 | Endocrine | AR |  | 10 |
|  | *ALMS1* | **27** | Retinitis pigmentosa |  | Ocular | AR | **Aldrees et al., 2019** | 1 |
| **25** | *ALPK1* | **28** | ROSAH syndrome | 614979 | Hematological, Immunological and Lymphatic | AD |  | 2 |
| **26** | *ANK3* | **29** | Inetellectual developmental disorder, type 37 | 615493 | Neurodevelopmental | AR |  | 1 |
| **27** | *ANOSMIN1* | **30** | Hypogonadotropic hypogonadism with or without anosmia (Kallmann syndrome, type 1) | 308700 | Renal and Genitourinary | XL |  | 1 |
| **28** | *ANTXR2* | **31** | Hyaline fibromatosis syndrome | 228600 | Connective tissue | AR |  | 1 |
| **29** | *APC* | **32** | Familial adenomatous polyposis, type 1 | 175100 | Cancer | AD |  | 4 |
| **30** | *APP* | **33** | Cerebral amyloid angiopathy | 605714 | Neurological | AD |  | 1 |
| **31** | *AR* | **34** | Spinal and bulbar muscular atrophy | 313200 | Neurological | XL |  | 1 |
|  | *AR* | **35** | Androgen insensitivity | 300068 | Renal and Genitourinary | XL |  | 1 |
| **32** | *ARG1* | **36** | Argininemia | 207800 | Metabolic | AR |  | 1 |
| **33** | *ARID1A* | **37** | Coffin-Siris syndrome, type 2 | 614607 | Neurodevelopmental | AD |  | 1 |
| **34** | *ARID1B* | **38** | Coffin-Siris syndrome, type 1 | 135900 | Neurodevelopmental | AD |  | 1 |
| **35** | *ARL3* | **39** | Distrofia de cono-baston |  | Ocular | AR | **Sheik et al., 2019** | 1 |
| **36** | *ARL6* | **40** | Retinitis pigmentosa, type 55 | 613575 | Ocular | AR |  | 2 |
|  | *ARL6* | **41** | Bardet-Biedl syndrome, type 3 | 600151 | Endocrine | AR |  | 3 |
|  | *ARL6* | **42** | Cone-rod dystrophy |  | Ocular | AR | **Zahid, S. et al. 2018.** | 1 |
| **37** | *ARMC9* | **43** | Joubert syndrome, type 30 | 617622 | Neurodevelopmental | AR |  | 1 |
| **38** | *ASH1L* | **44** | Intellectual developmental disorder, type 52 | 617796 | Neurodevelopmental | AD |  | 1 |
| **39** | *ASXL3* | **45** | Bainbridge-Ropers syndrome | 615485 | Neurodevelopmental | AD |  | 1 |
| **40** | *ATM* | **46** | Breast cancer, susceptibility to} | 114480 | Cancer | AD |  | 3 |
| **41** | *ATP1A3* | **47** | CAPOS syndrome | 601338 | Neurological | AD |  | 1 |
|  | *ATP1A3* | **48** | Alternating hemiplegia of childhood, type 2 | 614820 | Neurological | AD |  | 1 |
| **42** | *ATP2A2* | **49** | Darier disease | 124200 | Dermatological | AD |  | 1 |
| **43** | *ATP2C1* | **50** | Hailey-Hailey disease | 169600 | Dermatological | AD |  | 2 |
| **44** | *ATP7A* | **51** | Menkes disease | 309400 | Neurological | XL |  | 3 |
| **45** | *ATXN2* | **52** | Spinocerebellar ataxia, type 2 | 183090 | Neurological | AD |  | 2 |
| **46** | *ATXN3* | **53** | Machado-Joseph disease | 109150 | Neurological | AD |  | 1 |
| **47** | *AVPR2* | **54** | Diabetes insipidus, nephrogenic, type 1 | 304800 | Renal and Genitourinary | XL |  | 1 |
| **48** | *BBS1* | **55** | Bardet-Biedl syndrome, type 1 | 209900 | Endocrine | AR |  | 1 |
| **49** | *BBS10* | **56** | Bardet-Biedl syndrome, type 10 | 615987 | Endocrine | AR |  | 3 |
| **50** | *BBS5* | **57** | Bardet-Biedl syndrome, type 5 | 615983 | Endocrine | AR |  | 1 |
| **51** | *BBS7* | **58** | Bardet-Biedl syndrome, type 7 | 615984 | Endocrine | AR |  | 1 |
| **52** | *BBS9* | **59** | Bardet-Biedl syndrome, type 9 | 615986 | Endocrine | AR |  | 2 |
| **53** | *BCL11B* | **60** | Intellectual developmental disorder with dysmorphic facies, speech delay, and T-cell abnormalities | 618092 | Neurodevelopmental | AD |  | 1 |
| **54** | *BCOR* | **61** | Microphthalmia syndromic, type 2 | 300166 | Ocular | XL |  | 2 |
| **55** | *BEST1* | **62** | Macular dystrophy, vitelliform, type 2 | 153700 | Ocular | AD |  | 41 |
|  | *BEST1* | **63** | Retinitis pigmentosa, type 50 | 613194 | Ocular | AD |  | 1 |
|  | *BEST1* | **64** | Vitreoretinochoroidopathy | 193220 | Ocular | AD |  | 3 |
|  | *BEST1* | **65** | Bestrophinopathy | 611809 | Ocular | AR |  | 2 |
| **56** | *BLM* | **66** | Bloom syndrome | 210900 | Dermatological | AR |  | 2 |
| **57** | *BRCA1* | **67** | Breast-ovarian cancer, familial, type 1 | 604370 | Cancer | AD |  | 4 |
| **58** | *BRCA2* | **68** | {Breast-ovarian cancer, familial, type 2} | 612555 | Cancer | AD |  | 5 |
| **59** | *BRIP1* | **69** | Breast cancer, early onset, susceptibility | 114480 | Cancer | AD |  | 2 |
| **60** | *BTD* | **70** | Biotinidase deficiency | 253260 | Metabolic | AR |  | 1 |
| **61** | *BTK* | **71** | Agammaglobulinemia, type 1 | 300755 | Hematological, Immunological and Lymphatic | XL |  | 2 |
| **62** | *CABP4* | **72** | Cone-rod synaptic disorder, congenital nonprogressive | 610427 | Ocular | AR |  | 1 |
| **63** | *CACNA1A* | **73** | Migraine, familial hemiplegic, type 1, with progressive cerebellar ataxia | 141500 | Neurological | AD |  | 4 |
|  | *CACNA1A* | **74** | Spinocerebellar ataxia, type 6 | 183086 | Neurological | AD |  | 1 |
| **64** | *CACNA1C* | **75** | Neurodevelopmental disorder with hypotonia, language delay, and skeletal defects with or without seizures | 62009 | Neurodevelopmental | AD |  | 2 |
| **65** | *CACNA1F* | **76** | Cone-rod dystrophy, type 3 | 300476 | Ocular | XL |  | 3 |
|  | *CACNA1F* | **77** | Night blindness, congenital stationary (incomplete), type 2A | 300071 | Ocular | XL |  | 1 |
|  | *CACNA1F* | **78** | Aland Island eye disease | 300600 | Ocular | XL |  | 6 |
| **66** | *CAPN5* | **79** | Vitreoretinopathy, neovascular inflammatory | 193235 | Ocular | AD |  | 1 |
| **67** | *CAPN3* | **80** | Muscular dystrophy, limb-girdle, type 1 | 253600 | Muscular | AR |  | 1 |
| **68** | *CASP10* | **81** | Autoimmune lymphoproliferative syndrome, type II | 603909 | Hematological, Immunological and Lymphatic | AD |  | 1 |
| **69** | *CD46* | **82** | {Hemolytic uremic syndrome, atypical, susceptibility to, type 2} | 612922 | Renal and Genitourinary | AD |  | 1 |
| **70** | *CDC42BPB* | **83** | Chilton-Okur-Chung neurodevelopmental syndrome | 619841 | Neurodevelopmental | AD |  | 1 |
| **71** | *CDH23* | **84** | Usher syndrome, type ID/F | 601067 | Ocular | AR |  | 6 |
| **72** | *CDH3* | **85** | Ectodermal dysplasia, ectrodactyly, and macular dystrophy | 225280 | Dermatological | AR |  | 2 |
| **73** | *CDHR1* | **86** | Retinitis pigmentosa, type 65 | 613660 | Ocular | AR |  | 2 |
|  | *CDHR1* | **87** | Cone-rod dystrophy, type 15 | 613660 | Ocular | AR |  | 1 |
|  | *CDHR1* | **88** | Retinitis pigmentosa, type 65 | 613660 | Ocular | AR |  | 1 |
| **74** | *CDKL5* | **89** | Developmental and epileptic encephalopathy, type 2 | 300672 | Neurodevelopmental | AD |  | 1 |
| **75** | *CDKN2A* | **90** | {Melanoma-pancreatic cancer syndrome} | 606719 | Cancer | AD |  | 2 |
|  | *CDKN2A* | **91** | {Melanoma, cutaneous malignant, type 2} | 155601 | Cancer | AD |  | 6 |
| **76** | *CELF2* | **92** | Developmental and epileptic encephalopathy, type 97 | 619561 | Neurodevelopmental | AD |  | 1 |
| **77** | *CELSR1* | **93** | Lymphatic malformation, type 9 | 619319 | Hematological, Immunological and Lymphatic | AD |  | 1 |
| **78** | *CENPJ* | **94** | Microcephaly, type 6, primary | 608393 | Neurodevelopmental | AR |  | 1 |
| **79** | *CEP152* | **95** | Seckel syndrome 5 | 613823 | Neurodevelopmental | AR |  | 1 |
| **80** | *CEP250* | **96** | Retinitis pigmentosa |  | Ocular | AR | **Huang et al., 2019** | 1 |
| **81** | *CEP290* | **97** | Retnitis pigmentosa |  | Ocular | AR | **Testa et al., 2021** | 3 |
|  | *CEP290* | **98** | Leber congenital amaurosis, type 10 | 611755 | Ocular | AR |  | 4 |
|  | *CEP290* | **99** | Joubert syndrome, type 5 | 610188 | Ocular | AR |  | 1 |
| **82** | *CEP78* | **100** | Cone-rod dystrophy and hearing loss | 617236 | Ocular | AR |  | 1 |
| **83** | *CERKL* | **101** | Retinitis pigmentosa, type 26 | 608380 | Ocular | AR |  | 15 |
| **84** | *CFAP418* | **102** | Retinitis pigmentosa, type 64 | 614500 | Ocular | AR |  | 1 |
| **85** | *CHD3* | **103** | Snijders Blok-Campeau syndrome | 618205 | Neurodevelopmental | AD |  | 2 |
| **86** | *CHD7* | **104** | CHARGE syndrome | 214800 | Ocular | AD |  | 8 |
| **87** | *CHD8* | **105** | Intellectual developmental disorder with autism and macrocephaly | 615032 | Neurodevelopmental | AD |  | 1 |
| **88** | *CHN1* | **106** | Duane retraction syndrome, type 2 | 604356 | Ocular | AD |  | 1 |
| **89** | *CHM* | **107** | Choroideremia | 303100 | Ocular | XL |  | 40 |
| **90** | *CHRDL1* | **108** | Megalocornea, X-linked | 309300 | Ocular | XL |  | 1 |
| **91** | *CHST6* | **109** | Macular corneal dystrophy | 217800 | Ocular | AR |  | 2 |
| **92** | *CILK1* | **110** | {Epilepsy, juvenile myoclonic, susceptibility to, 10} | 617924 | Neurodevelopmental | AD |  | 1 |
| **93** | *CLCN4* | **111** | Raynaud-Claes syndrome | 300114 | Neurodevelopmental | XL |  | 1 |
| **94** | *CLCNKB* | **112** | Bartter syndrome, type 3 | 607364 | Renal and Genitourinary | AR |  | 1 |
| **95** | *CLN3* | **113** | Ceroid lipofuscinosis, neuronal, type 3 | 204200 | Neurological | AR |  | 2 |
|  | *CLN3* | **114** | Retinitis pigmentosa |  | Ocular | AR | **Wang et al., 2014** | 10 |
| **96** | *CLRN1* | **115** | Usher syndrome, type 3A | 276902 | Ocular | AR |  | 1 |
|  | *CLRN1* | **116** | Retinitis pigmentosa, type 61 | 614180 | Ocular | AR | . | 1 |
| **97** | *CNGA3* | **117** | Achromatopsia, type 2 | 216900 | Ocular | AR |  | 4 |
| **98** | *CNGB1* | **118** | Retinitis pigmentosa, type 45 | 613767 | Ocular | AR |  | 2 |
| **99** | *CNGB3* | **119** | Achromatopsia, type 3 | 262300 | Ocular | AR |  | 4 |
| **100** | *CNNM4* | **120** | Jalili syndrome | 217080 | Ocular | AR |  | 5 |
| **101** | *CNTNAP1* | **121** | Hypomyelinating neuropathy, congenital, type 3 | 618186 | Neurological | AR |  | 1 |
| **102** | *COL11A1* | **122** | Stickler syndrome, type II | 604841 | Connective tissue | AD |  | 2 |
| **103** | *COL18A1* | **123** | Knobloch syndrome, type 1 | 267750 | Ocular | AR |  | 2 |
| **104** | *COL1A2* | **124** | Osteogenesis imperfecta, type II | 166210 | Skeletal | AD |  | 1 |
| **105** | *COL2A1* | **125** | Stickler syndrome, Type I | 108300 | Connective tissue | AD |  | 5 |
|  | *COL2A1* | **126** | Stickler syndrome, nonsyndromic ocular | 609508 | Connective tissue | AD |  | 15 |
| **106** | *COL4A1* | **127** | Brain small vessel disease 1 with or without ocular anomalies | 175780 | Neurological | AD |  | 3 |
|  | *COL4A1* | **128** | ?Retinal arteries, tortuosity of | 180000 | Ocular | AD |  | 1 |
|  | *COL4A1* | **129** | COL4A1-Related Disorders |  | Cerebrovascular | AD | **Plaisier et al. 2016** | 1 |
| **107** | *COL4A3* | **130** | Alport syndrome, type 3A | 104200 | Renal and Genitourinary | AD |  | 4 |
| **108** | *COL4A4* | **131** | Alport syndrome, type 2 | 203780 | Renal and Genitourinary | AR |  | 1 |
| **109** | *COL5A1* | **132** | Ehlers-Danlos syndrome, classic type 1 | 130000 | Connective tissue | AD |  | 1 |
| **110** | *COL5A2* | **133** | Ehlers-Danlos syndrome, classic type 2 | 130010 | Connective tissue | AD |  | 1 |
| **111** | *COL6A1* | **134** | Ullrich muscular dystrophy | 254090 | Muscular | AD |  | 1 |
|  | *COL6A1* | **135** | Bethlem myopathy, type 1A | 158810 | Muscular | AD |  | 3 |
| **112** | *COL8A2* | **136** | Corneal dystrophy, Fuchs endothelial type 1 | 136800 | Ocular | AD |  | 1 |
| **113** | *COLQ* | **137** | Myasthenic syndrome, congenital, type 5 | 603034 | Neurological | AR |  | 1 |
| **114** | *COQ4* | **138** | Coenzyme Q10 deficiency, primary, type 7 | 616276 | Neurological | AR |  | 2 |
| **115** | *CP* | **139** | Aceruloplasminemia | 604290 | Neurological | AR |  | 1 |
| **116** | *CPAMD8* | **140** | Anterior segment dysgenesis, type 8 | 617319 | Ocular | AR |  | 2 |
| **117** | *CRB1* | **141** | Retinitis pigmentosa, nanophthalmos |  | Ocular | AR | **Zenteno et al., 2011** | 8 |
|  | *CRB1* | **142** | Leber congenital amaurosis, type 8 | 613835 | Ocular | AR |  | 17 |
|  | *CRB1* | **143** | Retinitis pigmentosa, type 12 | 600105 | Ocular | AR |  | 14 |
| **118** | *CREBBP* | **144** | Rubinstein-Taybi syndrome, type 1 | 180849 | Neurodevelopmental | AD |  | 1 |
| **119** | *CRX* | **145** | Cone-rod dystrophy. type 2 | 120970 | Ocular | AD |  | 7 |
| **120** | *CRYAA* | **146** | Cataract, congenital, type 9 | 604219 | Ocular | AD |  | 3 |
| **121** | *CRYBB2* | **147** | Cataract, congenital, type 3 | 601547 | Ocular | AD |  | 2 |
| **122** | *CRYGA* | **148** | Cataract, congenital |  | Ocular | AD | **Astiazarán et al., 2018** | 3 |
| **123** | *CRYGC* | **149** | Cataract, congenital, type 2 | 604307 | Ocular | AD |  | 2 |
| **124** | *CRYGD* | **150** | Cataract, congenital, type 4 | 115700 | Ocular | AD |  | 2 |
| **125** | *CSF1R* | **151** | Leukoencephalopathy, diffuse hereditary, with spheroids | 221820 | Neurological | AD |  | 1 |
| **126** | *CSNK2A1* | **152** | Okur-Chung neurodevelopmental syndrome | 617062 | Neurodevelopmental | AD |  | 2 |
| **127** | *CTSK* | **153** | Pycnodysostosis | 265800 | Skeletal | AR |  | 1 |
| **128** | *CYB5R3* | **154** | Methemoglobinemia, type I | 250800 | Hematological, Immunological and Lymphatic | AR |  | 3 |
| **129** | *CYP1B1* | **155** | Glaucoma, type 3A, primary open angle, congenital, juvenile or adult onset | 231300 | Ocular | AR |  | 26 |
|  | *CYP1B1* | **156** | Anterior segment dysgenesis, type 6 | 617315 | Ocular | AR |  | 3 |
| **130** | *CYP21A2* | **157** | Adrenal hyperplasia, congenital, due to 21-hydroxylase deficiency | 201910 | Metabolic | AR |  | 2 |
| **131** | *CYP2UI* | **158** | Spastic paraplegia, type 56 | 615030 | Neurological | AR |  | 1 |
| **132** | *CYP4V2* | **159** | Bietti crystalline corneoretinal dystrophy | 210370 | Ocular | AR |  | 1 |
| **133** | *DGUOK* | **160** | Progressive external ophthalmoplegia with mitochondrial DNA deletions, autosomal recessive, type 4 | 617070 | Neurological | AR |  | 1 |
| **134** | *DHTKD1* | **161** | ?Charcot-Marie-Tooth disease, axonal, type 2Q | 615025 | Neurological | AD |  | 1 |
| **135** | *DLG3* | **162** | Intellectual developmental disorder, type 90 | 300850 | Neurodevelopmental | XL |  | 1 |
| **136** | *DLL3* | **163** | Spondylocostal dysostosis, type 1 | 277300 | Skeletal | AR |  | 1 |
| **137** | *DMD* | **164** | Duchenne muscular dystrophy | 310200 | Muscular | XL |  | 3 |
| **138** | *DMPK* | **165** | Myotonic dystrophy, type 1 | 160900 | Neurological | AD |  | 11 |
| **139** | *DNAH5* | **166** | Ciliary dyskinesia, primary, type 3, with or without situs inversus | 608644 | Cardiovascular | AR |  | 2 |
| **140** | *DNAJB2* | **167** | Neuronopathy, distal hereditary motor, type 5 | 614881 | Neurological | AR |  | 1 |
| **141** | *DNMT3A* | **168** | Tatton-Brown-Rahman syndrome | 615879 | Neurodevelopmental | AD |  | 1 |
| **142** | *DOCK8* | **169** | Hyper-IgE syndrome, type 2 | 243700 | Hematological, Immunological and Lymphatic | AR |  | 1 |
| **143** | *DRAM2* | **170** | Cone-rod dystrophy, type 21 | 616502 | Ocular | AR |  | 3 |
| **144** | *DSG2* | **171** | Arrhythmogenic right ventricular dysplasia, type 10 | 610193 | Cardiovascular | AD |  | 2 |
| **145** | *DSP* | **172** | Arrhythmogenic right ventricular dysplasia, type 8 | 607450 | Cardiovascular | AD |  | 2 |
| **146** | *DYNC1I2* | **173** | Neurodevelopmental disorder with microcephaly and structural brain anomalies | 618492 | Neurodevelopmental | AR |  | 1 |
| **147** | *DYSF* | **174** | Muscular dystrophy, limb-girdle, type 2 | 253601 | Muscular | AR |  | 1 |
| **148** | *EBP* | **175** | Chondrodysplasia punctata | 302960 | Skeletal | XL |  | 2 |
| **149** | *EEF1A2* | **176** | Developmental and epileptic encephalopathy, type 33 | 616409 | Neurodevelopmental | AD |  | 1 |
| **150** | *EFEMP1* | **177** | Doyne Honeycomb retinal dystrophy | 126600 | Ocular | AD |  | 14 |
| **151** | *EFNB1* | **178** | Craniofrontonasal dysplasia | 304110 | Skeletal | XL |  | 5 |
| **152** | *EFTUD2* | **179** | Mandibulofacial dysostosis, Guion-Almeida type | 610536 | Skeletal | AD |  | 1 |
| **153** | *EHMT1* | **180** | Kleefstra syndrome, type 1 | 610253 | Neurodevelopmental | AD |  | 2 |
| **154** | *ELOVL5* | **181** | Spinocerebellar ataxia, type 38 | 615957 | Neurological | AD |  | 1 |
| **155** | *EPHA2* | **182** | Cataract, congenital, type 6 | 116600 | Ocular | AD |  | 4 |
| **156** | *EYA1* | **183** | Branchiootorenal syndrome, type 1, with or without cataracts | 113650 | Renal and Genitourinary | AD |  | 1 |
| **157** | *EYS* | **184** | Retinitis pigmentosa, type 25 | 602772 | Ocular | AR |  | 25 |
| **158** | *FA2H* | **185** | Spastic paraplegia, type 35 | 612319 | Neurological | AR |  | 1 |
| **159** | *FAM111B* | **186** | Poikiloderma, hereditary fibrosing, with tendon contractures, myopathy, and pulmonary fibrosis | 615704 | Dermatological | AD |  | 2 |
| **160** | *FAM161A* | **187** | Retinitis pigmentosa, type 28 | 606068 | Ocular | AR |  | 5 |
| **161** | *FANCA* | **188** | Fanconi anemia, complementation group A | 227650 | Hematological, Immunological and Lymphatic | AR |  | 1 |
| **162** | *FBLN5* | **189** | Macular degeneration, age-related, type 3 | 608895 | Ocular | AD |  | 1 |
| **163** | *FBN1* | **190** | Ectopia lentis, familial | 129600 | Ocular | AD |  | 11 |
|  | *FBN1* | **191** | Weill-Marchesani syndrome, type 2 | 608328 | Skeletal | AD |  | 1 |
|  | *FBN1* | **192** | Marfan syndrome | 154700 | Connective tissue | AD |  | 18 |
| **164** | *FBP1* | **193** | Fructose-1,6-bisphosphatase deficiency | 229700 | Metabolic | AR |  | 2 |
| **165** | *FDXR* | **194** | Multiple mitochondrial dysfunctions syndrome, type 9B | 620887 | Neurodevelopmental | AR |  | 1 |
| **166** | *FGFR1* | **195** | Encephalocraniocutaneous lipomatosis, somatic mosaic | 613001 | Dermatological | AD (somatic) |  | 1 |
| **167** | *FGFR2* | **196** | Apert syndrome | 101200 | Skeletal | AD |  | 1 |
|  | *FGFR2* | **197** | Crouzon Syndrome | 123500 | Skeletal | AD |  | 6 |
|  | *FGFR2* | **198** | Saethre-Chotzen syndrome | 101400 | Skeletal | AD |  | 1 |
| **168** | *FGFR3* | **199** | Hypochondroplasia | 146000 | Skeletal | AD |  | 2 |
| **169** | *FGF10* | **200** | LADD syndrome, type 3 | 620193 | Ocular | AD |  | 1 |
| **170** | *FKRP* | **201** | Muscular dystrophy-dystroglycanopathy (congenital with or without impaired intellectual development), type B, 5 | 606612 | Neurodevelopmental | AR |  | 1 |
| **171** | *FKTN* | **202** | Muscular dystrophy-dystroglycanopathy (congenital with brain and eye anomalies), type A, 4 | 253800 | Muscular | AR |  | 1 |
| **172** | *FLNA* | **203** | Otopalatodigital syndrome, type I | 311300 | Skeletal | XL |  | 1 |
| **173** | *FOXC1* | **204** | Anterior segment dysgenesis, type 3 | 601631 | Ocular | AD |  | 10 |
|  | *FOXC1* | **205** | Axenfeld-Rieger syndrome, type 3 | 602482 | Ocular | AD |  | 17 |
|  | *FOXC1* | **206** | De Hauwere Syndrome |  | Cardiovascular | AD | **Reis et al., 2023** | 1 |
| **174** | *FOXE3* | **207** | Anterior segment dysgenesis, type 2 | 610256 | Ocular | AR |  | 21 |
| **175** | *FOXG1* | **208** | Rett syndrome, congenital variant | 613454 | Neurodevelopmental | AD |  | 2 |
| **176** | *FOXL2* | **209** | Blepharophimosis, epicanthus inversus, and ptosis, types 1 and 2 | 110100 | Ocular | AD |  | 24 |
| **177** | *FOXP1* | **210** | Intellectual developmental disorder with language impairment with or without autistic features | 613670 | Neurodevelopmental | AD |  | 1 |
| **178** | *FREM1* | **211** | Manitoba oculotrichoanal syndrome | 248450 | Ocular | AR |  | 1 |
| **179** | *FTL* | **212** | Hyperferritinemia-cataract syndrome | 600886 | Hematological, Immunological and Lymphatic | AD |  | 2 |
| **180** | *FXN* | **213** | Friedreich ataxia | 229300 | Neurological | AR |  | 1 |
| **181** | *FZD4* | **214** | Exudative vitreoretinopathy, type 1 | 133780 | Ocular | AD |  | 1 |
| **182** | *G6PD* | **215** | Anemia, congenital, nonspherocytic hemolytic, type 1, G6PD deficient | 300908 | Hematological, Immunological and Lymphatic | XL |  | 4 |
| **183** | *GABBR2* | **216** | Developmental and epileptic encephalopathy, type 59 | 617904 | Neurodevelopmental | AD |  | 1 |
| **184** | *GABRA2* | **217** | Developmental and epileptic encephalopathy, type 78 | 618557 | Neurodevelopmental | AD |  | 1 |
| **185** | *GALC* | **218** | Krabbe disease | 245200 | Neurodevelopmental | AR |  | 2 |
| **186** | *GALK1* | **219** | Galactokinase deficiency | 230200 | Metabolic | AR |  | 4 |
| **187** | *GALNS* | **220** | Mucopolysaccharidosis, type IVA | 253000 | Metabolic | AR |  | 1 |
| **188** | *GAN* | **221** | Giant axonal neuropathy-1 | 256850 | Neurological | AR |  | 1 |
| **189** | *GANAB* | **222** | Polycystic kidney disease, type 3 | 600666 | Renal and Genitourinary | AD |  | 1 |
| **190** | *GBA* | **223** | Parkinson disease, late-onset, susceptibility to | 168600 | Neurological | AD |  | 1 |
|  | *GBA* | **224** | Gaucher disease, type I | 230800 | Hematological, Immunological and Lymphatic | AR |  | 1 |
| **191** | *GBF1* | **225** | Charcot-Marie-Tooth disease, axonal, type 2GG | 606483 | Neurological | AD |  | 2 |
| **192** | *GCDH* | **226** | Glutaric aciduria, type I | 231670 | Metabolic | AR |  | 5 |
| **193** | *GCK* | **227** | MODY, type II | 125851 | Metabolic | AD |  | 3 |
| **194** | *GDAP1* | **228** | Charcot-Marie-Tooth disease, axonal, type 2K | 607831 | Neurological | AD |  | 1 |
| **195** | *GDF6* | **229** | Microphthalmia isolated, type 4 | 613094 | Ocular | AD |  | 4 |
| **196** | *GFAP* | **230** | Alexander disease | 203450 | Neurological | AD |  | 1 |
| **197** | *GJA1* | **231** | Oculodentodigtal dysplasia | 164200 | Ocular | AD |  | 4 |
| **198** | *GJA3* | **232** | Cataract, congenital, type 14 | 601885 | Ocular | AD |  | 1 |
| **199** | *GJA8* | **233** | Cataract, congenital, type 1 | 116200 | Ocular | AD |  | 2 |
| **200** | *GJB2* | **234** | Keratitis-ichthyosis-deafness syndrome | 148210 | Dermatological | AD |  | 2 |
| **201** | *GMPPA* | **235** | Alacrima, Achalasia, and impaired intellectual development syndrome | 615510 | Neurodevelopmental | AR |  | 2 |
| **202** | *GNAL* | **236** | Dystonia, type 25 | 615073 | Neurological | AD |  | 2 |
| **203** | *GNAS* | **237** | McCune-Albright syndrome, somatic mosaic | 174800 | Endocrine | somatic |  | 1 |
| **204** | *GNAT1* | **238** | Night blindness, congenital stationary, type 1G | 616389 | Ocular | AR |  | 2 |
| **205** | *GNB1* | **239** | Intellectual developmental disorder, type 42 | 616973 | Neurodevelopmental | AD |  | 1 |
| **206** | *GPR143* | **240** | Ocular albinism, type I | 300500 | Ocular | XL |  | 6 |
| **207** | *GPR179* | **241** | Night blindness, congenital stationary (complete), type 1E | 614565 | Ocular | AR |  | 1 |
| **208** | *GRHPR* | **242** | Hyperoxaluria, primary, type II | 260000 | Renal and Genitourinary | AR |  | 1 |
| **209** | *GRIA2* | **243** | Neurodevelopmental disorder with language impairment and behavioral abnormalities | 618917 | Neurodevelopmental | AD |  | 2 |
| **210** | *GSDME* | **244** | Deafness, type 5 | 600994 | Neurological | AD |  | 1 |
| **211** | *GSN* | **245** | Amyloidosis, Finnish type | 105120 | Ocular | AD |  | 3 |
| **212** | *GUCA1A* | **246** | Cone-rod dystrophy, type 14 | 602093 | Ocular | AD |  | 3 |
| **213** | *GUCA1B* | **247** | Retinitis pigmentosa, type 48 | 613827 | Ocular | AD |  | 1 |
| **214** | *GUCY2D* | **248** | Leber congenital amaurosis, type 1 | 204000 | Ocular | AR |  | 19 |
|  | *GUCY2D* | **249** | Cone-rod dystrophy, type 6 | 601777 | Ocular | AD |  | 9 |
| **215** | *GZF1* | **250** | Joint laxity, short stature, and myopia | 617662 | Connective tissue | AR |  | 6 |
| **216** | *HBA1* | **251** | Erythrocytosis, familial, type 7 | 617981 | Hematological, Immunological and Lymphatic | AD |  | 1 |
| **217** | *HBA2* | **252** | Erythrocytosis, familial, type 7 | 617981 | Hematological, Immunological and Lymphatic | AD |  | 1 |
|  | *HBA2* | **253** | Thalassemia, alpha- | 604131 | Hematological, Immunological and Lymphatic | AR |  | 2 |
| **218** | *HEXA* | **254** | Tay-Sachs disease | 272800 | Neurodevelopmental | AR |  | 1 |
| **219** | *HFE* | **255** | Hemochromatosis, type 1 | 235200 | Hematological, Immunological and Lymphatic | AR |  | 1 |
| **220** | *HGSNAT* | **256** | Retinitis pigmentosa, type 73 | 616544 | Ocular | AR |  | 1 |
|  | *HGSNAT* | **257** | Mucopolysaccharidosis type IIIC (Sanfilippo C) | 252930 | Neurodevelopmental | AR |  | 3 |
| **221** | *HMBS* | **258** | Porphyria, acute intermittent | 176000 | Hematological, Immunological and Lymphatic | AD |  | 1 |
| **222** | *HMCN1* | **259** | Macular degeneration, age-related, type 1 | 603075 | Ocular | AD |  | 1 |
| **223** | *HNF1A* | **260** | MODY, type III | 600496 | Endocrine | AD |  | 3 |
| **224** | *HNRNPH2* | **261** | Intellectual developmental disorder, Bain type | 300986 | Neurodevelopmental | XL |  | 1 |
| **225** | *HTT* | **262** | Huntington disease | 143100 | Neurological | AD |  | 1 |
| **226** | *IARS2* | **263** | Cataracs, growth hormone deficiency, sensory neuropathy, sensoryneural hearing loss, and skeletal dysplasia | 616007 | Neurodevelopmental | AR |  | 1 |
| **227** | *IDH3B* | **264** | Retinitis pigmentosa, type 46 | 612572 | Ocular | AR |  | 1 |
| **228** | *IFT140* | **265** | Retinitis pigmentosa, type 80 | 617781 | Ocular | AR |  | 8 |
| **229** | *IMPG1* | **266** | Retinitis pigmentosa, type 91 | 153870 | Ocular | AD |  | 4 |
| **230** | *IMPG2* | **267** | Retinitis pigmentosa, type 56 | 613581 | Ocular | AR |  | 4 |
|  |  | **268** | Macular dystrophy, vitelliform, 5 | 616152 | Ocular | AD |  | 9 |
| **231** | *IRF2BPL* | **269** | Neurodevelopmental disorder with regression, abnormal movements, loss of speech, and seizures | 618088 | Neurodevelopmental | AD |  | 1 |
| **232** | *JAG1* | **270** | Alagille syndrome, type 1 | 118450 | Ocular | AD |  | 4 |
| **233** | *KCNB1* | **271** | Developmental and epileptic encephalopathy, type 26 | 616056 | Neurodevelopmental | AD |  | 1 |
| **234** | *KCNC2* | **272** | Developmental and epileptic encephalopathy, type 103 | 619913 | Neurodevelopmental | AD |  | 1 |
| **235** | *KCND3* | **273** | Spinocerebellar ataxia, type 19 | 607346 | Neurodevelopmental | AD |  | 1 |
| **236** | *KCNH2* | **274** | Long QT syndrome, type 2 | 613688 | Cardiovascular | AD |  | 4 |
|  | *KCNH2* | **275** | Short QT syndrome, type 1 | 609620 | Cardiovascular | AD |  | 1 |
| **237** | *KCNJ11* | **276** | Hyperinsulinemic hypoglycemia, familial, type 2 | 601820 | Endocrine | AD |  | 1 |
| **238** | *KCNJ2* | **277** | Andersen cardiodysrhythmic periodic paralysis | 170390 | Cardiovascular | AD |  | 1 |
| **239** | *KCNQ1* | **278** | Long QT syndrome, type 1 | 192500 | Cardiovascular | AD |  | 1 |
| **240** | *KCNQ2* | **279** | Developmental and epileptic encephalopathy, type 7 | 613720 | Neurodevelopmental | AD |  | 2 |
| **241** | *KCNV2* | **280** | Retinal cone dystrophy, type 3B | 610356 | Ocular | AR |  | 2 |
| **242** | *KDM1A* | **281** | Cleft palate, psychomotor retardation, and distinctive facial features | 616728 | Neurodevelopmental | AD |  | 1 |
| **243** | *KDM5A* | **282** | KDM5A-Associated Disorders |  | Neurodevelopmental | AD | **El Hayek et al., 2020** | 1 |
| **244** | *KDM5B* | **283** | KDM5B-Associated Disorders |  | Neurodevelopmental | AD | **Borroto et al., 2024** | 1 |
| **245** | *KDM5C* | **284** | Intellectual developmental disorder, X-linked syndromic, Claes-Jensen type | 300534 | Neurodevelopmental | XL |  | 1 |
| **246** | *KERA* | **285** | Cornea plana, type 2 | 217300 | Ocular | AR |  | 3 |
| **247** | *KIAA1549* | **286** | Retinitis pigmentosa, type 86 | 618613 | Ocular | AR |  | 1 |
| **248** | *KIF1A* | **287** | NESCAV syndrome | 614255 | Neurodevelopmental | AD |  | 1 |
| **249** | *KIF11* | **288** | Microcephaly with or without chorioretinopathy, lymphedema or impaired intellectual developmen | 152950 | Neurodevelopmental | AD |  | 3 |
| **250** | *KIF21A* | **289** | Fibrosis of extraocular muscles congenital, type 1 | 135700 | Ocular | AD |  | 6 |
| **251** | *KIF2A* | **290** | Cortical dysplasia, complex, with other brain malformations, type 3 | 615411 | Neurodevelopmental | AD |  | 1 |
| **252** | *KIF5A* | **291** | Spastic paraplegia, type 10 | 604187 | Neurological | AD |  | 1 |
| **253** | *KIF7* | **292** | Acrocallosal syndrome | 200990 | Neurodevelopmental | AR |  | 1 |
| **254** | *KIZ* | **293** | Retinitis pigmentosa, type 69 | 615780 | Ocular | AR |  | 1 |
| **255** | *KMT2A* | **294** | Wiedemann-Steiner syndrome | 605130 | Neurodevelopmental | AD |  | 4 |
| **256** | *KMT2D* | **295** | Kabuki syndrome, type 1 | 147920 | Neurodevelopmental | AD |  | 3 |
| **257** | *KRAS* | **296** | Oculoectodermal syndrome, somatic | 600268 | Dermatological | somatic |  | 3 |
|  | *KRAS* | **297** | Schimmelpenning-Feuerstein-Mims syndrome, somatic mosaicism | 163200 | Dermatological | somatic |  | 3 |
|  | *KRAS* | **298** | Noonan syndrome, type 3 | 609942 | Neurodevelopmental | AD |  | 1 |
| **258** | *KRT10* | **299** | Epidermolytic hyperkeratosis, type 2A | 620150 | Dermatological | AD |  | 1 |
| **259** | *LAMA2* | **300** | Muscular dystrophy, congenital, merosin deficient or partially deficient | 607855 | Muscular | AR |  | 2 |
| **260** | *LBR* | **301** | Reynolds syndrome | 613471 | Hematological, Immunological and Lymphatic | AD |  | 1 |
| **261** | *LCA5* | **302** | Leber congenital amaurosis, type 5 | 604537 | Ocular | AR |  | 3 |
| **262** | *LDLR* | **303** | Hypercholesterolemia, familial, type 1 | 143890 | Metabolic | AD |  | 5 |
| **263** | *LMNA* | **304** | Hutchinson-Gilford progeria | 176670 | Cardiovascular | AD |  | 1 |
| **264** | *LOXL1* | **305** | Exfoliation syndrome, susceptibility | 177650 | Ocular | AD |  | 1 |
| **265** | *LPIN1* | **306** | Myoglobinuria, acute recurrent | 268200 | Muscular | AR |  | 1 |
| **266** | *LPL* | **307** | Lipase deficiency, combined | 246650 | Metabolic | AR |  | 1 |
| **267** | *LRAT* | **308** | Leber congenital amaurosis, type 14 | 613341 | Ocular | AR |  | 2 |
| **268** | *LRP2* | **309** | Donnai-Barrow syndrome | 222448 | Ocular | AR |  | 2 |
| **269** | *LRP5* | **310** | Exudative vitreoretinopathy, type 4 | 601813 | Ocular | AR |  | 4 |
|  | *LRP5* | **311** | Osteoporosis-pseudoglioma syndrome | 259770 | Skeletal | AR |  | 1 |
| **270** | *LRRK2* | **312** | Parkinson disease 8 | 607060 | Neurological | AD |  | 3 |
| **271** | *LYST* | **313** | Chediak-Higashi syndrome | 214500 | Dermatological | AR |  | 1 |
| **272** | *MAPK8IP3* | **314** | Neurodevelopmental disorder with or without variable brain abnormalities | 618443 | Neurodevelopmental | AD |  | 1 |
| **273** | *MCPH1* | **315** | Hereditary Breast Cancer Susceptibility |  | Cancer | AD | **Mantere et al., 2016** | 1 |
| **274** | *MECP2* | **316** | Rett syndrome | 312750 | Neurodevelopmental | XL |  | 2 |
| **275** | *MED12* | **317** | MED12-Related Disorders |  | Neurodevelopmental | XL | **Lyons et al., 2021** | 2 |
| **276** | *MEFV* | **318** | Familial Mediterranean fever | 134610 | Hematological, Immunological and Lymphatic | AD |  | 3 |
| **277** | *MERTK* | **319** | Retinitis pigmentosa, type 38 | 613862 | Ocular | AR |  | 5 |
| **278** | *MFN2* | **320** | Hereditary motor and sensory neuropathy, type VIA | 601152 | Neurological | AD |  | 2 |
| **279** | *MFRP* | **321** | Nanophthalmos, type 2 | 609549 | Ocular | AR |  | 1 |
|  | *MFRP* | **322** | Nanophthalmos, retinitis pigmentosa, foveoschisis, optic disk drusen |  | Ocular | AR | **Ayala-Ramirez et al., 2006** | 6 |
| **280** | *MFSD8* | **323** | Macular dystrophy with central cone involvement | 616170 | Ocular | AR |  | 1 |
|  | *MFSD8* | **324** | Ceroid lipofuscinosis, neuronal, type 7 | 610951 | Neurodevelopmental | AR |  | 1 |
| **281** | *MITF* | **325** | Waardenburg syndrome, type 2A | 193510 | Dermatological | AD |  | 3 |
| **282** | *MKKS* | **326** | Bardet-Biedl syndrome, type 6 | 605231 | Endocrine | AR |  | 2 |
| **283** | *MSH6* | **327** | Lynch syndrome, type 5 | 614350 | Cancer | AD |  | 1 |
| **284** | *MSX2* | **328** | Parietal foramina, type 1 | 168500 | Skeletal | AD |  | 1 |
| **285** | *MT-ATP6* | **329** | Leigh syndrome, mithocondrial | 500017 | Neurological | MT |  | 0 |
|  | *MT-ATP6* | **330** | Neuropathy, ataxia, and retinitis pigmentosa | 551500 | Neurological | MT |  | 5 |
| **286** | *MT-ND4* | **331** | Leber Hereditary Optic Neuropathy; LHON | 535000 | Ocular | MT |  | 5 |
| **287** | *MT-ND6* | **332** | Leber Hereditary Optic Neuropathy; LHON | 535000 | Ocular | MT |  | 2 |
| **288** | *MT-TK* | **333** | Myoclonic Epilepsy with Ragged Red Fibers | 545000 | Muscular | MT |  | 1 |
| **289** | *MT-TL1* | **334** | Mitochondrial Myopathy, Encephalopathy, Lactic Acidosis, And Stroke-Like Episodes; MELAS | 540000 | Neurological | MT |  | 3 |
| **290** | *MT-TV* | **335** | Charcot-Marie-Tooth Disease, Axonal, Mitochondrial; CMTMA | 500013 | Neurological | MT |  | 1 |
| **291** | *MTMT1* | **336** | Myopathy, centronuclear | 310400 | Muscular | XL |  | 1 |
| **292** | *MTOR* | **337** | Smith-Kingsmore syndrome | 616638 | Neurodevelopmental | AD |  | 1 |
| **293** | *MTSS2* | **338** | Intellectual developmental disorder with ocular anomalies and distinctive facial features | 620086 | Neurodevelopmental | AD |  | 1 |
| **294** | *MYBPC3* | **339** | Cardiomyopathy, hypertrophic, type 4 | 115197 | Cardiovascular | AD |  | 4 |
| **295** | *MYH2* | **340** | Congenital myopathy, type 6, with ophthalmoplegia | 605637 | Muscular | AD |  | 1 |
| **296** | *MYO7A* | **341** | Usher syndrome, type 1B | 276900 | Ocular | AR |  | 10 |
| **297** | *MYOC* | **342** | Glaucoma, primary open angle, type 1A | 137750 | Ocular | AD |  | 37 |
| **298** | *MYRF* | **343** | Cardiac-urogenital syndrome | 618280 | Cardiovascular | AD |  | 2 |
| **299** | *MYTL1* | **344** | Intellectual developmental disorder, type 39 | 616521 | Neurodevelopmental | AD |  | 1 |
| **300** | *NAA10* | **345** | Microphthalmia syndromic, type 1 | 309800 | Ocular | XL |  | 4 |
| **301** | *NAA15* | **346** | Intellectual developmental disorder, type 50, with behavioral abnormalities | 617787 | Neurodevelopmental | AD |  | 3 |
| **302** | *NBEA* | **347** | Neurodevelopmental disorder with or without early-onset generalized epilepsy | 619157 | Neurodevelopmental | AD |  | 1 |
| **303** | *NDP* | **348** | Norrie disease | 310600 | Ocular | XL |  | 39 |
| **304** | *NF1* | **349** | Neurofibromatosis-Noonan syndrome | 601321 | Dermatological | AD |  | 1 |
|  | *NF1* | **350** | Neurofibromatosis, type 1 | 162200 | Dermatological | AD |  | 3 |
| **305** | *NF2* | **351** | Schwannomatosis, vestibular | 101000 | Neurological | AD |  | 1 |
| **306** | *NIPBL* | **352** | Cornelia de Lange syndrome, type 1 | 122470 | Neurodevelopmental | AD |  | 1 |
| **307** | *NLRP7* | **353** | Hydatidiform mole, recurrent, type 1 | 231090 | Renal and Genitourinary | AR |  | 1 |
| **308** | *NMNAT1* | **354** | Leber congenital amaurosis, type 9 | 608553 | Ocular | AR |  | 2 |
| **309** | *NOD2* | **355** | Blau syndrome | 186580 | Hematological, Immunological and Lymphatic | AD |  | 1 |
| **310** | *NPC1* | **356** | Niemann-Pick disease, type C1 | 257220 | Metabolic | AR |  | 1 |
| **311** | *NPHS2* | **357** | Nephrotic syndrome, type 2 | 600995 | Renal and Genitourinary | AR |  | 1 |
| **312** | *NR2E3* | **358** | Retinitis pigmentosa, type 37 | 611131 | Ocular | AD |  | 8 |
| **313** | *NRL* | **359** | Retinitis pigmentosa, type 27 | 613750 | Ocular | AD |  | 5 |
| **314** | *NSD1* | **360** | Sotos syndrome | 117550 | Neurodevelopmental | AD |  | 5 |
| **315** | *NSD2* | **361** | Rauch-Steindl syndrome | 619695 | Neurodevelopmental | AD |  | 4 |
| **316** | *NTRK2* | **362** | Developmental and epileptic encephalopathy, type 58 | 617830 | Neurodevelopmental | AD |  | 1 |
| **317** | *NYX* | **363** | Night blindness, congenital stationary (complete), type 1A, | 310500 | Ocular | XL |  | 1 |
| **318** | *OAT* | **364** | Gyrate atrophy of choroid and retina | 258870 | Metabolic | AR |  | 10 |
| **319** | *OCA2* | **365** | Albinism, oculocutaneous, type II | 203200 | Dermatological | AR |  | 6 |
| **320** | *OCRL* | **366** | Lowe syndrome | 309000 | Neurodevelopmental | XL |  | 2 |
| **321** | *OPA1* | **367** | Optic atrophy, type 1 | 165500 | Ocular | AD |  | 6 |
|  | *OPA1* | **368** | Behr syndrome | 210000 | Ocular | AR |  | 1 |
| **322** | *OPTN* | **369** | Amyotrophic lateral sclerosis, type12, with or witout frontotemporal dementia | 613435 | Neurological | AD |  | 1 |
| **323** | *OTX2* | **370** | Microphthalmia syndromic, type 5 | 610125 | Ocular | AD |  | 5 |
|  | *OTX2* | **371** | Retinal dystrophy, early-onset, with or without pituitary dysfunction | 610125 | Ocular | AD |  | 1 |
| **324** | *PABPN1* | **372** | Oculopharyngeal muscular dystrophy | 164300 | Neurological | AD |  | 185 |
| **325** | *PACS1* | **373** | Schuurs-Hoeijmakers syndrome | 615009 | Neurodevelopmental | AD |  | 3 |
| **326** | *PALB2* | **374** | {Breast-ovarian cancer, familial, susceptibility to, 5} | 620442 | Cancer | AD |  | 3 |
| **327** | *PANK2* | **375** | Neurodegeneration with brain iron accumulation, type 1 | 234200 | Neurodevelopmental | AR |  | 14 |
| **328** | *PAX3* | **376** | Wardenburg syndrome, type 1 | 193500 | Dermatological | AD |  | 7 |
| **329** | *PAX6* | **377** | Aniridia | 106210 | Ocular | AD |  | 31 |
| **330** | *PCARE* | **378** | Retinitis pigmentosa, type 54 | 613428 | Ocular | AR |  | 14 |
|  | *PCARE* | **379** | Cone-rod dystrophy, type 23 | 613428 | Ocular | AR |  | 5 |
| **331** | *PCDH15* | **380** | Usher syndrome, type 1D/F digenic | 601067 | Ocular | AR |  | 2 |
| **332** | *PDE6A* | **381** | Retinitis pigmentosa, type 43 | 613810 | Ocular | AR |  | 6 |
| **333** | *PDE6B* | **382** | Retinitis pigmentosa, type 40 | 613801 | Ocular | AR |  | 5 |
| **334** | *PDE6H* | **383** | Retinitis pigmentosa | 610024 | Ocular | AD |  | 1 |
| **335** | *PDHA1* | **384** | Pyruvate dehydrogenase E1-alpha defiency | 312170 | Metabolic | XL |  | 1 |
| **336** | *PEX1* | **385** | Heimler syndrome, type 1 | 234580 | Metabolic | AR |  | 2 |
| **337** | *PEX26* | **386** | Peroxisome biogenesis disorder, type 7A (Zellweger) | 614872 | Metabolic | AR |  | 1 |
| **338** | *PEX6* | **387** | Heimler syndrome, type 2 | 616617 | Metabolic | AR |  | 1 |
| **339** | *PIEZO1* | **388** | Lymphatic malformation, type 6 | 616843 | Hematological, Immunological and Lymphatic | AR |  | 1 |
| **340** | *PIK3R1* | **389** | SHORT syndrome | 269880 | Skeletal | AD |  | 1 |
| **341** | *PIKFYVE* | **390** | Corneal fleck dystrophy | 121850 | Ocular | AD |  | 2 |
| **342** | *PITPNM3* | **391** | Cone-rod dystrophy, type 5 | 600977 | Ocular | AD |  | 1 |
| **343** | *PITX2* | **392** | Axenfeld-Rieger syndrome, type 1 | 180500 | Ocular | AD |  | 6 |
| **344** | *PKD1* | **393** | Polycystic kidney disease, type 1 | 173900 | Renal and Genitourinary | AD |  | 9 |
| **345** | *PKHD1* | **394** | Polycystic kidney disease, type 4, with or without hepatic disease | 263200 | Renal and Genitourinary | AR |  | 1 |
| **346** | *PLP1* | **395** | Pelizaeus-Merzbacher disease | 312080 | Neurodevelopmental | XL |  | 3 |
| **347** | *POC1B* | **396** | Cone-rod dystrophy, type 20 | 615973 | Ocular | AR |  | 9 |
| **348** | *POLA1* | **397** | Pigmentary disorder, reticulate, with systemic manifestations | 301220 | Dermatological | XL |  | 1 |
| **349** | *POLG* | **398** | Progressive external ophthalmoplegia, type 1 | 157640 | Ocular | AD |  | 5 |
| **350** | *POLR2A* | **399** | Neurodevelopmental disorder with hypotonia and variable intellectual and behavioral abnormalities | 618603 | Neurodevelopmental | AD |  | 1 |
| **351** | *POLR3A* | **400** | Leukodystrophy, hypomyelinating, type 7, with or without oligodontia and/or hypogonadotropic hypogonadism | 607694 | Neurodevelopmental | AR |  | 1 |
| **352** | *POLRMT* | **401** | Combined oxidative phosphorylation deficiency, type 55 | 619743 | Neurological | AR |  | 1 |
| **353** | *PPARG* | **402** | Lipodystrophy, familial partial, type 3 | 604367 | Endocrine | AD |  | 1 |
| **354** | *PPP1CB* | **403** | Noonan syndrome-like disorder with loose anagen hair, type 2 | 617506 | Neurodevelopmental | AD |  | 1 |
| **355** | *PPT1* | **404** | Ceroid lipofuscinosis, neuronal, type 1 | 256730 | Neurological | AR |  | 1 |
| **356** | *PRCD* | **405** | Retinitis pigmentosa, type 36 | 610599 | Ocular | AR |  | 2 |
| **357** | *PRDM13* | **406** | Macular dystrophy, retinal, type 1, North Carolina type, MCDR1 | 136550 | Ocular | AD |  | 4 |
| **358** | *PREPL* | **407** | Myasthenic syndrome, congenital, type 22 | 616224 | Neurodevelopmental | AR |  | 1 |
| **359** | *PRF1* | **408** | Hemophagocytic lymphohistiocytosis, familial, type 2 | 603553 | Hematological, Immunological and Lymphatic | AR |  | 1 |
| **360** | *PRKAR1A* | **409** | Carney complex, type 1 | 160980 | Dermatological | AD |  | 1 |
| **361** | *PRKN* | **410** | Parkinson disease, juvenile, type 2 | 600116 | Neurological | AR |  | 1 |
| **362** | *PRMT7* | **411** | Short stature, brachydactyly, intellectual developmental disability, and seizures | 617157 | Neurodevelopmental | AR |  | 2 |
| **363** | *PRNP* | **412** | Creutzfeldt-Jakob disease | 123400 | Neurological | AD |  | 1 |
| **364** | *PROM1* | **413** | Stargardt disease, type 4 | 603786 | Ocular | AD |  | 1 |
|  | *PROM1* | **414** | Retinitis pigmentosa, type 41 | 612095 | Ocular | AR |  | 2 |
|  | *PROM1* | **415** | Macular dystrophy, retinal, type 2 | 608051 | Ocular | AD |  | 5 |
|  | *PROM1* | **416** | Cone-rod dystrophy, type 12 | 612657 | Ocular | AR |  | 11 |
| **365** | *PRPF3* | **417** | Retinosis pigmentosa, type 18 | 601414 | Ocular | AD |  | 11 |
| **366** | *PRPF31* | **418** | Retinitis pigmentosa, type 11 | 600138 | Ocular | AD |  | 24 |
| **367** | *PRPF6* | **419** | Retinitis pigmentosa, type 60 | 613983 | Ocular | AD |  | 1 |
| **368** | *PRPF8* | **420** | Retinitis pigmentosa, type 13 | 600059 | Ocular | AD |  | 2 |
| **369** | *PRPH12* | **421** | Retinitis pigmentosa, type 7 | 608133 | Ocular | AD |  | 1 |
| **370** | *PRPH2* | **422** | Macular dystrophy, patterned, type 1 | 169150 | Ocular | AD |  | 6 |
| **371** | *PRR12* | **423** | Neurocoular syndrome | 619539 | Neurodevelopmental | AD |  | 1 |
| **372** | *PRSS56* | **424** | Microphthalmia, isolated, type 6 | 613517 | Ocular | AR |  | 10 |
| **373** | *PSAP* | **425** | Metachromatic leukodystrophy due to SAP-b deficiency | 249900 | Neurodevelopmental | AR |  | 1 |
| **374** | *PSEN1* | **426** | Alzheimer disease, type 3, with or without spastic paraparesis | 607822 | Neurological | AD |  | 1 |
| **375** | *PSTPIP1* | **427** | Pyogenic sterile arthritis, pyoderma gangrenosum, and acne | 604416 | Dermatological | AD |  | 1 |
| **376** | *PTCH1* | **428** | Gorlin Goltz syndrome | 109400 | Neurodevelopmental | AD |  | 1 |
| **377** | *PTEN* | **429** | Cowden syndrome, type 1 | 158350 | Neurodevelopmental | AD |  | 3 |
| **378** | *PTPN11* | **430** | Noonan syndrome, type 1 | 163950 | Neurodevelopmental | AD |  | 5 |
| **379** | *PUM1* | **431** | Neurodevelopmental disorder with motor abnormalities, and facial dysmorphism | 620719 | Neurodevelopmental | AD |  | 1 |
| **380** | *PURA* | **432** | Neurodevelopmental disorder with neonatal respiratory insufficiency, hypotonia, and feeding difficulties | 616158 | Neurodevelopmental | AD |  | 1 |
| **381** | *PXDN* | **433** | Anterior segment dysgenesis, type 7 | 269400 | Ocular | AR |  | 4 |
| **382** | *RAB11B* | **434** | Neurodevelopmental disorder with ataxic gait, absent speech, and decreased cortical white matter | 617807 | Neurodevelopmental | AD |  | 1 |
| **383** | *RAB3GAP1* | **435** | Warburg micro syndrome, type 1 | 600118 | Neurodevelopmental | AR |  | 1 |
| **384** | *RAD51C* | **436** | {Breast-ovarian cancer, familial, susceptibility to, 3} | 613399 | Cancer | AD |  | 1 |
| **385** | *RAX* | **437** | Microphthalmia, Syndromic, type 16 | 611038 | Ocular | AR |  | 2 |
| **386** | *RB1* | **438** | Retinoblastoma | 180200 | Cancer | AD |  | 4 |
| **387** | *RBP3* | **439** | Retinitis pigmentosa, type 66 | 615233 | Ocular | AR |  | 1 |
| **388** | *RDH12* | **440** | Retinitis pigmentosa |  | Ocular | AD | **Sarkar et al., 2020.** | 2 |
|  | *RDH12* | **441** | Leber congenital amaurosis, type 13 | 612712 | Ocular | AR |  | 32 |
| **389** | *RDH5* | **442** | Fundus albipunctatus | 136880 | Ocular | AD |  | 5 |
| **390** | *REEP6* | **443** | Retinitis pigmentosa, type 77 | 617304 | Ocular | AR |  | 1 |
| **391** | *RHO* | **444** | Retinitis pigmentosa, type 4 | 613731 | Ocular | AD |  | 26 |
| **392** | *RNASEH2A* | **445** | Aicardi-Goutieres syndrome, type 4 | 610333 | Neurodevelopmental | AR |  | 1 |
| **393** | *RNF170* | **446** | Ataxia, sensory, type 1 | 608984 | Neurological | AD |  | 1 |
| **394** | *RNU6-2* | **447** | Retinitis pigmentosa |  | Ocular | AD | **Quinodoz et al. 2025** | 1 |
| **395** | *ROM1* | **448** | Retinitis pigmentosa, type 7 | 608133 | Ocular | AD |  | 1 |
| **396** | *RORA* | **449** | Intellectual developmental disorder with or without epilepsy or cerebellar ataxia | 618060 | Neurodevelopmental | AD |  | 1 |
| **397** | *RP1* | **450** | Retinitis pigmentosa, type 1 | 180100 | Ocular | AD |  | 18 |
| **398** | *RP1L1* | **451** | Retinitis pigmentosa, type 88 | 618826 | Ocular | AR |  | 1 |
| **399** | *RP2* | **452** | Retinitis pigmentosa, type 2 | 312600 | Ocular | XL |  | 21 |
| **400** | *RPE65* | **453** | Leber congenital amaurosis, type 2 | 204100 | Ocular | AR |  | 22 |
|  | *RPE65* | **454** | Retinitis pigmentosa, type 20 | 613794 | Ocular | AR |  | 3 |
| **401** | *RPGR* | **455** | Retinitis pigmentosa, type 3 | 312610 | Ocular | XL |  | 30 |
|  | *RPGR* | **456** | Macular degeneration, atrophic | 300834 | Ocular | XL |  | 3 |
| **402** | *RPGRIP1* | **457** | Cone-rod dystrophy, type 13 | 608194 | Ocular | AR |  | 1 |
|  | *RPGRIP1* | **458** | Leber congenital amaurosis, type 6 | 613826 | Ocular | AR |  | 16 |
| **403** | *RRM2B* | **459** | Progressive external ophthalmoplegia with mitochondrial DNA deletions, type 5 | 613077 | Neurological | AD |  | 1 |
| **404** | *RS1* | **460** | Retinoschisis, type 1, juvenile | 312700 | Ocular | XL |  | 31 |
| **405** | *RTEL1* | **461** | Dyskeratosis congenita, type 5 | 615190 | Dermatological | AR |  | 1 |
| **406** | *RTN41IP1* | **462** | Optic atrophy 10 with or without ataxia, impaired intellectual development and seizures | 616732 | Neurodevelopmental | AR |  | 1 |
| **407** | *RUNX2* | **463** | Cleidocranial dysplasia | 119600 | Skeletal | AD |  | 1 |
| **408** | *RYR1* | **464** | Congenital myopathy, type 1A | 117000 | Neurological | AD |  | 3 |
|  | *RYR1* | **465** | Malignant hyperthermia susceptibility 1 | 145600 | Neurological | AD |  | 2 |
| **409** | *SAG* | **466** | Retinitis pigmentosa, type 96 | 620228 | Ocular | AD |  | 3 |
| **410** | *SALL4* | **467** | Duane-radial ray syndrome | 607323 | Skeletal | AD |  | 2 |
| **411** | *SAMD7* | **468** | Macular dystrophy with or without cone dysfunction | 620762 | Ocular | AR |  | 1 |
| **412** | *SCLT1* | **469** | Retinal dystrophy, non syndromic |  | Ocular | AR | **Sangermano et al., 2025** | 1 |
| **413** | *SCN11A* | **470** | Episodic pain syndrome, familial, type 3 | 615552 | Neurological | AD |  | 4 |
| **414** | *SCN1A* | **471** | Dravet syndrome | 607208 | Neurodevelopmental | AD |  | 6 |
|  | *SCN1A* | **472** | Developmental and epileptic encephalopathy, type 6B, non-Dravet | 619317 | Neurodevelopmental | AD |  | 1 |
| **415** | *SCN2A* | **473** | Developmental and epileptic encephalopathy, type 11 | 613721 | Neurodevelopmental | AD |  | 1 |
| **416** | *SCN4A* | **474** | Paramyotonia congenita | 168300 | Neurological | AD |  | 1 |
| **417** | *SCN5A* | **475** | Long QT syndrome, type 3 | 603830 | Cardiovascular | AD |  | 1 |
|  | *SCN5A* | **476** | Brugada syndrome, type 1 | 601144 | Cardiovascular | AD |  | 1 |
| **418** | *SCN9A* | **477** | SCN9A Neuropathic Pain Syndromes |  | Neurological | AD | **Hisama et al., 2020** | 3 |
| **419** | *SDHD* | **478** | Pheochromocytoma/paraganglioma syndrome, type 1 | 168000 | Endocrine | AD |  | 1 |
| **420** | *SLC34A1* | **479** | Nephrolithiasis/osteoporosis, hypophosphatemic, 1 | 612286 | Metabolic | AD |  | 1 |
| **421** | *SERPINA1* | **480** | Alpha-1-Antitrypsin Deficiency | 613490 | Metabolic | AR |  | 1 |
| **422** | *SETD1A* | **481** | Neurodevelopmental disorder with speech impairment and dysmorphic facies | 619056 | Neurodevelopmental | AD |  | 1 |
| **423** | *SETD1B* | **482** | Intellectual developmental disorder with seizures and language delay | 619000 | Neurodevelopmental | AD |  | 1 |
| **424** | *SETD5* | **483** | Intellectual developmental disorder, type 23 | 615761 | Neurodevelopmental | AD |  | 1 |
| **425** | *SETX* | **484** | Spinocerebellar ataxia, with axonal neuropathy, type 2 | 606002 | Neurological | AR |  | 1 |
| **426** | *SGCE* | **485** | Dystonia, type 11, myoclonic | 159900 | Neurological | AD |  | 1 |
| **427** | *SGSH* | **486** | Mucopolysaccharidosis type IIIA (Sanfilippo A) | 252900 | Neurodevelopmental | AR |  | 1 |
| **428** | *SHANK3* | **487** | Phelan-McDermid syndrome | 606232 | Neurodevelopmental | AD |  | 1 |
| **429** | *SHH* | **488** | Holoprosencephaly, type 3 | 142945 | Neurodevelopmental | AD |  | 1 |
| **430** | *SIX1* | **489** | Branchiootic syndrome, type 3 | 608389 | Skeletal | AD |  | 1 |
| **431** | *SLC25A15* | **490** | Hyperornithinemia-hyperammonemia-homocitrullinemia syndrome | 238970 | Neurodevelopmental | AR |  | 3 |
| **432** | *SLC45A2* | **491** | Albinism, oculocutaneous, type IV | 606574 | Dermatological | AR |  | 1 |
| **433** | *SLC4A11* | **492** | Corneal endothelial dystrophy | 217700 | Ocular | AR |  | 1 |
| **434** | *SLC6A1* | **493** | Myoclonic-atonic epilepsy | 616421 | Neurological | AD |  | 1 |
| **435** | *SMAD6* | **494** | Radioulnar synostosis, nonsyndromic | 179300 | Skeletal | AD |  | 1 |
| **436** | *SMC3* | **495** | Cornelia de Lange syndrome, type 3 | 610759 | Neurodevelopmental | AD |  | 1 |
| **437** | *SMPD1* | **496** | Niemann-Pick disease type A | 257200 | Metabolic | AR |  | 1 |
| **438** | *SNRNP200* | **497** | Retinitis pigmentosa, type 33 | 610359 | Ocular | AD |  | 14 |
| **439** | *SOHLH1* | **498** | Ovarian dysgenesis, type 5 | 617690 | Renal and Genitourinary | AR |  | 4 |
| **440** | *SOS1* | **499** | Noonan syndrome, type 4 | 610733 | Neurodevelopmental | AD |  | 8 |
| **441** | *SOX10* | **500** | Waardenburg syndrome, type 2E, with or without neurologic involvement | 611584 | Dermatological | AD |  | 2 |
| **442** | *SOX2* | **501** | Microphthalmia, Syndromic, type 3 | 206900 | Ocular | AD |  | 4 |
|  | *SOX2* | **502** | Optic nerve hypoplasia and abnormalities of the central nervous system | 206900 | Ocular | AD |  | 1 |
| **443** | *SOX4* | **503** | Coffin-Siris syndrome, type 10 | 618506 | Neurodevelopmental | AD |  | 1 |
| **444** | *SOX9* | **504** | 46XY sex reversal, type 10 | 616425 | Renal and Genitourinary | AD |  | 1 |
| **445** | *SPATA7* | **505** | Leber congenital amaurosis, type 3 | 604232 | Ocular | AR |  | 5 |
|  | *SPATA7* | **506** | Retinitis pigmentosa, type 94 | 604232 | Ocular | AR |  | 3 |
| **446** | *SPECC1L* | **507** | Teebi hypertelorism syndrome, type 1 | 145420 | Skeletal | AD |  | 1 |
| **447** | *SPG7* | **508** | Spastic paraplegia, type 7 | 607259 | Neurological | AR |  | 1 |
| **448** | *SPTAN1* | **509** | Developmental and epileptic encephalopathy, type 5 | 613477 | Neurodevelopmental | AD |  | 2 |
| **449** | *SPTB* | **510** | Spherocytosis, type 2 | 616649 | Hematological, Immunological and Lymphatic | AD |  | 3 |
| **450** | *SPTLC1* | **511** | Neuropathy, hereditary sensory and autonomic, type IA | 162400 | Neurological | AD |  | 1 |
| **451** | *SREBF1* | **512** | Mucoepithelial dysplasia, hereditary | 158310 | Dermatological | AD |  | 2 |
| **452** | *SRRM2* | **513** | Intellectual developmental disorder, type 72 | 620439 | Neurodevelopmental | AD |  | 2 |
| **453** | *STAMBP* | **514** | Neurodevelopmental disorder associated to STAMBP |  | Neurodevelopmental | AR | **Hu et al., 2022** | 2 |
| **454** | *STK11* | **515** | Peutz-Jeghers syndrome | 175200 | Cancer | AD |  | 2 |
| **455** | *STS* | **516** | Icthyosis | 308100 | Dermatological | XL |  | 1 |
| **456** | *STXBP1* | **517** | Developmental and epileptic encephalopathy, type 4 | 612164 | Neurodevelopmental | AD |  | 1 |
| **457** | *SUCLG1* | **518** | Mitochondrial DNA depletion syndrome, type 9 (encephalomyopathic type with methylmalonic aciduria) | 245400 | Neurodevelopmental | AR |  | 1 |
| **458** | *SUOX* | **519** | Sulfite oxidase deficiency | 272300 | Neurodevelopmental | AR |  | 1 |
| **459** | *SYNE1* | **520** | Emery-Dreifuss muscular dystrophy, type 4 | 612998 | Neurological | AD |  | 1 |
| **460** | *TACSTD2* | **521** | Corneal dystrophy, gelatinous drop-like | 204870 | Ocular | AR |  | 10 |
| **461** | *TBC1D24* | **522** | Developmental and epileptic encephalopathy, type 16 | 615338 | Neurodevelopmental | AR |  | 7 |
|  | *TBC1D24* | **523** | Myoclonic epilepsy, infantile, familial | 605021 | Neurodevelopmental | AR |  | 1 |
| **462** | *TBCD* | **524** | Encephalopathy, progressive, early-onset, with brain atrophy and thin corpus callosum | 617193 | Neurodevelopmental | AR |  | 1 |
| **463** | *TBL1XR1* | **525** | Intellectual developmental disorder, type 41 | 616944 | Neurodevelopmental | AD |  | 1 |
| **464** | *TBP* | **526** | Spinocerebellar ataxia, type 17 | 607136 | Neurological | AD |  | 2 |
| **465** | *TBX18* | **527** | Congenital anomalies of kidney and urinary tract, type 2 | 143400 | Renal and Genitourinary | AD |  | 2 |
| **466** | *TBXAS1* | **528** | Ghosal hematodiaphyseal syndrome | 231095 | Hematological, Immunological and Lymphatic | AR |  | 1 |
| **467** | *TCF20* | **529** | Developmental delay with variable intellectual impairment and behavioral abnormalities | 618430 | Neurodevelopmental | AD |  | 1 |
| **468** | *TEK* | **530** | Glaucoma, type 3, primary congenital, E | 617272 | Ocular | AD |  | 16 |
| **469** | *TFAP2A* | **531** | Branchiooculofacial syndrome | 113620 | Ocular | AD |  | 7 |
| **470** | *TFAP2B* | **532** | Char syndrome | 169100 | Cardiovascular | AD |  | 1 |
| **471** | *TGFBI* | **533** | Corneal dystrophy, Reis Bucklers type | 608470 | Ocular | AD |  | 11 |
|  | *TGFBI* | **534** | Corneal dystrophy, Avelino type Granular type 2 | 607541 | Ocular | AD |  | 12 |
|  | *TGFBI* | **535** | Corneal dystrophy, Granular type 1 | 121900 | Ocular | AD |  | 35 |
|  | *TGFBI* | **536** | Corneal dystrophy, Lattice type 1 | 122200 | Ocular | AD |  | 20 |
|  | *TGFBI* | **537** | Corneal dystrophy, Thiel Benke type | 602082 | Ocular | AD |  | 1 |
| **472** | *TGM1* | **538** | Ichthyosis, congenital, type 1 | 242300 | Dermatological | AR |  | 13 |
| **473** | *TIMP3* | **539** | Sorsby fundus dystrophy | 136900 | Ocular | AD |  | 1 |
| **474** | *TK2* | **540** | Mitochondrial DNA depletion syndrome, type 2 (myopathic type) | 609560 | Muscular | AR |  | 1 |
| **475** | *TMC6* | **541** | Epidermodysplasia verruciformis | 226400 | Dermatological | AR |  | 2 |
| **476** | *TMEM127* | **542** | {Pheochromocytoma, susceptibility to} | 171300 | Endocrine | AD |  | 1 |
| **477** | *TMEM231* | **543** | Joubert syndrome, type 20 | 614970 | Neurodevelopmental | AR |  | 2 |
| **478** | *TMEM67* | **544** | COACH syndrome, type 1 | 216360 | Neurodevelopmental | AR |  | 1 |
| **479** | *TNFRSF13B* | **545** | Inmunodeficiency, common variable, type 2 | 240500 | Hematological, Immunological and Lymphatic | AD |  | 1 |
| **480** | *TNFRSF1A* | **546** | Periodic fever, familial | 142680 | Hematological, Immunological and Lymphatic | AD |  | 1 |
| **481** | *TNNI3* | **547** | Cardiomyopathy, hypertrophic, type 7 | 613690 | Cardiovascular | AD |  | 1 |
| **482** | *TNNI3K* | **548** | Cardiac conduction disease with/without dilated cardiomiopathy | 616117 | Cardiovascular | AD |  | 1 |
| **483** | *TOPORS* | **549** | Retinitis pigmentosa, type 31 | 609923 | Ocular | AD |  | 10 |
| **484** | *TP53* | **550** | Li-Fraumeni syndrome | 151623 | Cancer | AD |  | 1 |
| **485** | *TP63* | **551** | Ectrodactyly, ectodermal dysplasia, and cleft lip/palate syndrome, type 3 | 604292 | Skeletal | AD |  | 5 |
|  | *TP63* | **552** | ADULT syndrome | 103285 | Skeletal | AD |  | 1 |
| **486** | *TRRAP* | **553** | Developmental delay with or without dysmorphic facies and autism | 618454 | Neurodevelopmental | AD |  | 3 |
| **487** | *TSC1* | **554** | Tuberous sclerosis, type 1 | 191100 | Cancer | AD |  | 1 |
| **488** | *TTC8* | **555** | Bardet-Biedl syndrome, type 8 | 615985 | Endocrine | AR |  | 1 |
| **489** | *TTPA* | **556** | Ataxia with isolated vitamin E deficiency | 277460 | Neurological | AR |  | 2 |
| **490** | *TTR* | **557** | Amyloidosis, hereditary, transthyretin-related | 105210 | Cardiovascular | AD |  | 6 |
|  | *TTR* | **558** | Müller cell dystrophy |  | Ocular | AD | **Dalma-Weiszhausz et al., 2022** | 1 |
| **491** | *TUBA1A* | **559** | Lissencephaly 3 | 611603 | Neurodevelopmental | AD |  | 1 |
| **492** | *TUBB* | **560** | Cortical dysplasia, complex, with other brain malformations, type 6 | 615771 | Neurodevelopmental | AD |  | 1 |
| **493** | *TUBB3* | **561** | Fibrosis of extraocular muscles, congenital, type 3A | 600638 | Ocular | AD |  | 1 |
| **494** | *TULP1* | **562** | Retinitis pigmentosa, type 14 | 600132 | Ocular | AR |  | 3 |
|  | *TULP1* | **563** | Cone-rod dystrophy |  | Ocular | AR | **Boulanger-Scemama et al., 2015** | 1 |
|  | *TULP1* | **564** | Leber congenital amaurosis, type 15 | 613843 | Ocular | AR |  | 9 |
| **495** | *TWIST2* | **565** | Barber-Say syndrome | 209885 | Dermatological | AD |  | 1 |
| **496** | *TYR* | **566** | Albinism, oculocutaneous, type IA | 203100 | Dermatological | AR |  | 1 |
| **497** | *UBIAD1* | **567** | Corneal dystrophy, Schnyder type | 121800 | Ocular | AD |  | 5 |
| **498** | *UBQLN2* | **568** | Amyotrophic lateral sclerosis, type 15, with or without frontotemporal dementia | 300857 | Neurological | XL |  | 1 |
| **499** | *UGT1A1* | **569** | Crigler-Najjar syndrome, type I | 218800 | Metabolic | AR |  | 3 |
| **500** | *UNC119* | **570** | Cone-rod dystrophy, type 24 | 620342 | Ocular | AD |  | 1 |
| **501** | *USH1C* | **571** | Usher syndrome, type 1C | 276904 | Ocular | AR |  | 1 |
| **502** | *USH2A* | **572** | Usher syndrome type 2A | 276901 | Ocular | AR |  | 46 |
|  | *USH2A* | **573** | Retinitis pigmentosa, type 39 | 613809 | Ocular | AR |  | 51 |
| **503** | *VHL* | **574** | Von Hippel-Lindau syndrome | 193300 | Cancer | AD |  | 40 |
| **504** | *VMA21* | **575** | Myopathy, with excessive autophagy | 310440 | Muscular | XL |  | 1 |
| **505** | *VPS13B* | **576** | Cohen syndrome | 216550 | Neurodevelopmental | AR |  | 2 |
| **506** | *VSX1* | **577** | Craneofacial anomalies and anterior segment dysgenesis syndrome | 614195 | Ocular | AD |  | 1 |
| **507** | *VSX2* | **578** | Micropthalmia, isolated, type 2 | 610093 | Ocular | AR |  | 1 |
| **508** | *VWA8* | **579** | Retinitis pigmentosa, type 97 | 620422 | Ocular | AD |  | 4 |
| **509** | *WDR19* | **580** | Retinitis pigmentosa |  | Ocular | AR | **Coussa et al., 2013** | 3 |
| **510** | *WDR45* | **581** | Neurodegeneration with brain iron accumulation, type 5 | 300894 | Neurodevelopmental | XL |  | 2 |
| **511** | *WFS1* | **582** | Wolfram syndrome, type1 | 222300 | Ocular | AR |  | 11 |
| **512** | *ZC4H2* | **583** | Wieacker-Wolff syndrome, female-restricted | 301041 | Neurodevelopmental | XL |  | 1 |
| **513** | *ZNF469* | **584** | Brittle cornea syndrome, type 1 | 229200 | Ocular | AR |  | 3 |
| **514** | 1p31.1-p31.3 deletion | **585** | Chromosome 1p31 deletion syndrome |  | Neurodevelopmental | AD | **Genovese et al., 2015** | 1 |
| **515** | 1p36.33 deletion | **586** | Chromosome 1p36 deletion syndrome, distal | 607872 | Neurodevelopmental | AD |  | 1 |
| **516** | 1q21 deletion | **587** | Chromosome 1q21.1 deletion syndrome | 612474 | Neurodevelopmental | AD |  | 1 |
| **517** | 2q24.1-q24.2 deletion | **588** | Chromosome 2q24.1-q24.2 deletion syndrome |  | Neurodevelopmental | AD | **Palumbo et al., 2012** | 1 |
| **518** | 3q29 deletion | **589** | Chromosome 3q29 microdeletion syndrome | 609425 | Neurodevelopmental | AD |  | 1 |
| **519** | 4p16.3 deletion | **590** | Chromosome 4p16.3 deletion syndrome | 194190 | Neurodevelopmental | AD |  | 1 |
| **520** | 8q21.3-8q22.1 deletion | **591** | Chromosome 8q22.1 deletion syndrome |  | Neurodevelopmental | AD | **Overhoff et al., 2014** | 1 |
| **521** | 11p13 deletion | **592** | Wilms tumor, aniridia, genitourinary anomalies and impaired intellectual development syndrome | 194072 | Renal and Genitourinary | AD |  | 1 |
| **522** | 13q32.1 deletion | **593** | Microcoria, congenital | 156600 | Ocular | AD |  | 1 |
| **523** | 15q11.2 deletion | **594** | Chromosome 15q11.2 deletion syndrome | 615656 | Neurodevelopmental | AD |  | 1 |
| **524** | 15q11.2-q13 deletion (maternal) | **595** | Angelman syndrome | 105830 | Neurodevelopmental | AD |  | 1 |
|  | 15q11.2-q13 UPD (paternal) |  |  |  |  |  |  | 1 |
| **525** | 15q11.2-q13 deletion (paternal) | **596** | Prader-Willi syndrome | 176270 | Neurodevelopmental | AD |  | 1 |
|  | 15q11.2-q13 UPD (maternal) |  |  |  |  |  |  | 1 |
| **526** | 15q11.2-q13 duplication | **597** | Chromosome 15q11-q13 duplication syndrome | 608636 | Neurodevelopmental | AD |  | 1 |
| **527** | 16p11.2 deletion | **598** | Chromosome 16p11.2 deletion syndrome | 611913 | Neurodevelopmental | AD |  | 1 |
| **528** | 16p13.11 duplication | **599** | Chromosome 16p13.11 duplication syndrome |  | Neurodevelopmental | AD | **Allach El Khattabi et al., 2020** | 1 |
| **529** | 17p12 duplication | **600** | Charcot-Marie-Tooth disease, type 1A | 118220 | Neurological | AD |  | 2 |
| **530** | 17p13.3 deletion | **601** | Chromosome 17p13.3 deletion syndrome | 247200 | Neurodevelopmental | AD |  | 1 |
| **531** | 18p11.32 deletion | **602** | Chromosome 18p deletion syndrome | 146390 | Neurodevelopmental | AD |  | 1 |
| **532** | 18q22.1 deletion | **603** | Chromosome 18q deletion syndrome | 601808 | Neurodevelopmental | AD |  | 1 |
| **533** | 22q11.2 deletion | **604** | Chromosome 22q11.2 deletion syndrome | 188400 | Neurodevelopmental | AD |  | 2 |
| **534** | Xp22.31 deletion | **605** | Chromosome Xp22.31 deletion syndrome |  | Neurodevelopmental | XL | **Ma et al., 2020** | 1 |
|  |  |  |  |  |  |  |  | **n=2579** |
